# Tumor dormancy and relapse: understanding the molecular mechanisms of cancer recurrence

**DOI:** 10.1186/s40779-025-00595-2

**Published:** 2025-02-11

**Authors:** Muhammad Tufail, Can-Hua Jiang, Ning Li

**Affiliations:** 1 Department of Oral and Maxillofacial Surgery, Center of Stomatology, Xiangya Hospital, Central South University, Changsha, 410008 China; 2 Institute of Oral Precancerous Lesions, Central South University, Changsha, 410008 China; 3 Research Center of Oral and Maxillofacial Tumor, Xiangya Hospital, Central South University, Changsha, 410008 China; 4 National Clinical Research Center for Geriatric Disorders, Xiangya Hospital, Central South University, Changsha, 410008 China

**Keywords:** Tumor dormancy, Cancer recurrence, Signaling pathways, Biomarkers, Therapeutic approaches

## Abstract

Cancer recurrence, driven by the phenomenon of tumor dormancy, presents a formidable challenge in oncology. Dormant cancer cells have the ability to evade detection and treatment, leading to relapse. This review emphasizes the urgent need to comprehend tumor dormancy and its implications for cancer recurrence. Despite notable advancements, significant gaps remain in our understanding of the mechanisms underlying dormancy and the lack of reliable biomarkers for predicting relapse. This review provides a comprehensive analysis of the cellular, angiogenic, and immunological aspects of dormancy. It highlights the current therapeutic strategies targeting dormant cells, particularly combination therapies and immunotherapies, which hold promise in preventing relapse. By elucidating these mechanisms and proposing innovative research methodologies, this review aims to deepen our understanding of tumor dormancy, ultimately facilitating the development of more effective strategies for preventing cancer recurrence and improving patient outcomes.

## Background

Cancer recurrence represents a significant challenge in oncology and contributes to elevated morbidity and mortality rates among patients [1]. Despite progress in early detection, surgical techniques, and adjuvant therapies, a considerable proportion of patients experience relapse following the initial period of remission. The phenomenon of cancer recurrence is multi-faceted, involving numerous biological processes and mechanisms that enable dormant cancer cells to evade treatment, survive in a quiescent state, and eventually reinitiate aggressive growth [2, 3]. Understanding these processes is essential for developing more effective strategies to prevent and manage recurrent diseases.

Tumor dormancy refers to a state in which cancer cells remain viable but non-proliferative, possessing the potential to reactivate and induce clinical relapse [4, 5]. Various factors, including genetic and epigenetic alterations, can exert an influence on this state [6, 7], interactions with the tumor microenvironment (TME), and dynamics of immune system [8]. The transition from dormancy to an active disease involves complex signaling pathways and environmental triggers that promote cell proliferation and survival [9, 10]. Comprehending these mechanisms is crucial for identifying biomarkers associated with dormancy and relapse, predicting the risk of recurrence, and developing targeted therapies aimed at preventing or treating recurrent diseases.

This review is needed to provide a more comprehensive and integrated analysis of tumor dormancy and cancer recurrence, despite the availability of other reviews on the subject. Many current reviews focus on isolated aspects of dormancy and relapse, leaving a gap in understanding the full spectrum of mechanisms involved [10, 11]. Our review aims to bridge this gap by offering a complete overview that encompasses the definitions, types, and underlying mechanisms of tumor dormancy and relapse.

Moreover, this review emphasizes key signaling pathways, their roles, and potential therapeutic targets. We investigate the mechanisms underlying tumor relapse, with a focus on dormant cell reactivation, genetic and epigenetic changes, as well as the TME. Clinical implications are also discussed, including diagnostic biomarkers, strategies for predicting recurrence risk, and therapeutic approaches. Furthermore, challenges in managing cancer recurrence along with future research directions are examined, highlighting innovative diagnostic tools and personalized medicine. The significance of this study lies in its potential to integrate current knowledge and advancements, ultimately improving patient outcomes while achieving long-term cancer remission, contributing to more effective interventions that reduce cancer recurrence.

## Types of dormancy

### Cellular dormancy

**Fig. 1:**
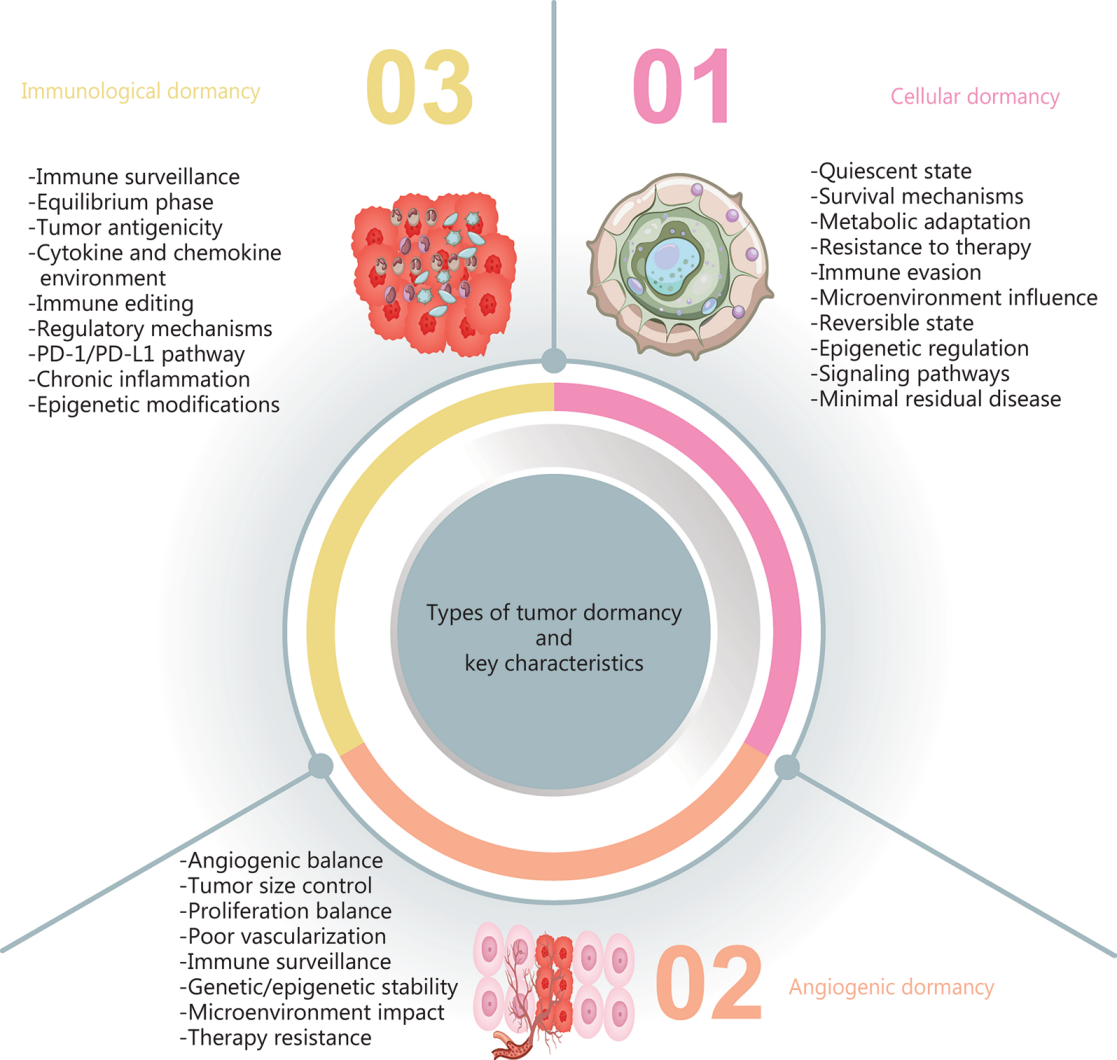
Mechanisms of tumor dormancy: cellular, angiogenic, and immunologic dormancy. Cellular dormancy, where cancer cells in a quiescent state. These cells are in a non-proliferative state and exhibit resistance to chemotherapy. Angiogenic dormancy, featuring a small tumor mass with limited blood vessels, indicates a lack of sufficient angiogenesis. Key features include balanced proliferation and apoptosis, as well as dependence on angiogenic factors. Immunological dormancy, showcasing interactions between cancer cells and immune cells (e.g., T cells, macrophages), highlighting immune surveillance. The immune system keeps tumor cells in check, involving immune checkpoints and cytokines to maintain dormancy

Tumor dormancy encompasses three primary types: cellular, angiogenic, and immunological dormancy, each characterized by distinct features (Fig. 1). Cellular dormancy refers to the reversible quiescent phase into which individual cancer cells can enter. During this phase, cells cease proliferation while remaining viable and metabolically active [12]. This state allows cancer cells to endure prolonged periods under adverse conditions, including those induced by cancer therapies.

Cell cycle arrest is a critical feature of cellular dormancy. Dormant cells typically exit the active cell cycle and enter the G0 phase, which is characterized as a non-dividing state [9]. This transition is regulated by a complex network of signaling pathways and molecular mechanisms. Key regulators include proteins known as cyclin-dependent kinase inhibitors, such as p21 and p27, which hinder the progression of the cell cycle and maintain cells in a dormant state [13].

A significant aspect of cellular dormancy is its reversibility [14]. Unlike permanent senescence, in which cells irreversibly lose their ability to proliferate, reversible senescence allows cells to temporarily exit the cell cycle and enter a dormant state. This state can be reversed, enabling cells to re-enter the cell cycle in response to specific environmental cues or therapeutic interventions [10, 15]. Reversible senescence contributes to tumor heterogeneity and therapeutic resistance, similar to other dormancy mechanisms such as diapause and persister cells [10, 15]. Diapause, commonly observed in embryos and certain adult organisms, involves a temporary suspension of development and metabolic activity that provides a survival advantage under adverse conditions [16]. Persister cells, a well-documented phenomenon in bacterial populations, represent a small subset of cells that evade antimicrobial treatment by entering a dormant-like state. Cancer researchers are increasingly recognizing this concept wherein persister cells can survive chemotherapy and subsequently drive relapse [16, 17].

Various factors, such as the restoration of blood supply or alterations in the extracellular matrix (ECM), can trigger the reactivation of dormant cells, particularly those in a state of reversible senescence. The reactivation is frequently accompanied by the upregulation of growth factors and cytokines that promote cell division and survival pathways. For example, when blood flow is restored or new vasculature forms to provide reoxygenation, dormant cells may be stimulated to resume proliferation [18]. Moreover, the binding of integrins and other cell surface receptors on dormant cancer cells to specific ECM components can activate signaling cascades that drive cell cycle re-entry. When disseminated dormant cancer cells encounter a novel ECM composition at distant sites, this ECM-receptor interaction can trigger their reactivation [19]. Additionally, research has revealed that the release of docetaxel-induced protumor cytokines interleukin-6 (IL-6) and granulocyte colony-stimulating factor (G-CSF) stimulate the outgrowth of dormant cancer cells both in vitro and in vivo. Single-cell transcriptomics and tumor stromal organoid models show that these reawakened cancer cells exhibit enhanced stemness, chemoresistance, and an altered TME, characterized by increased protumor immune signaling. IL-6 promotes cancer cell proliferation while G-CSF contributes to tumor immunosuppression. Targeting the mitogen-activated protein kinase (MEK) pathway offers a promising strategy for preventing escape from dormancy and recurrence through the inhibition of IL-6, G-CSF, as well as MEK/extracellular signal-regulated kinase (ERK) pathways using selumetinib prior to treatment with docetaxel [20].

Moreover, the survival of dormant cells under stress conditions is a critical determinant in cancer recurrence. Dormant cells often exhibit resistance to conventional therapies, which primarily target rapidly dividing cells [21]. This resistance can be attributed, in part, to their quiescent state, rendering them less susceptible to the mechanisms of action employed by these therapies. Additionally, dormant cells may activate survival pathways, such as the phosphoinositide 3-kinase/protein kinase B/mechanistic target of rapamycin (PI3K/Akt/mTOR) pathway, thereby enhancing their ability to endure therapeutic disruptions. For example, researchers have linked diminished PI3K/Akt signaling, which plays a key role in regulating various cellular processes, to dormancy markers such as the absence of Ki-67 and proliferating cell nuclear antigen, suggesting that it may also contribute to initiating the dormancy state [21]. A recent study indicates that stress hormones may stimulate dormant cancer cells that remain in the body after treatment, further strengthening their survival under stress conditions [22]. The experiments demonstrated that stress hormones triggered a cascade reaction in immune cells that led to the reactivation of dormant cancer cells.

Cellular dormancy also involves metabolic adaptations that facilitate cell survival during periods of low metabolic activity [23]. Several factors can influence tumor recurrence, including the composition of ECM, the effect of stromal cells, and nutrient availability [23]. Basically, dormancy phenotypes are characterized by reduced metabolic activity (hypometabolism), diminished nutrient intake, and decreased reproductive capacity. Under adverse environmental conditions, both normal and cancerous cells exhibit a wide range of states, ranging from quiescence to prolonged dormancy [24]. Dormant cells often switch to alternative metabolic pathways to conserve energy and maintain viability. For instance, they may enhance autophagy, a process that degrades and recycles cellular components, to fulfill their energy requirements. These metabolic changes are essential for the long-term survival of dormant cells in nutrient-deprived environments [25].

Understanding the mechanisms underlying cellular dormancy is indispensable for developing strategies to prevent cancer recurrence. Targeting the pathways that regulate both dormancy and reactivation may yield therapies capable of either eliminating dormant cells or maintaining them in a non-proliferative state. For instance, inhibiting specific signaling pathways involved in the maintenance of dormancy or its reactivation could effectively prevent dormant cells from re-entering the cell cycle and subsequently causing relapse [9, 10].

### Angiogenic dormancy

Angiogenic dormancy refers to a state in which tumor growth is restricted due to insufficient blood supply. In this condition, the tumor remains small and non-proliferative as it cannot induce the formation of new blood vessels, a process known as angiogenesis [26]. Angiogenesis is crucial for tumor growth and progression [27]. Tumors require a blood supply to obtain oxygen and nutrients that support their rapid proliferation. Without the development of new blood vessels, tumors are incapable of growing beyond a certain size, typically 1–2 mm in diameter [28]. An illustrative example can be observed in the rat insulin promoter 1 (RIP1-Tag2) transgenic mouse model, where autochthonous tumors arise in the pancreatic islets due to the expression of SV40 T antigen. However, only around 4% of these tumors become angiogenic and exceed microscopic size after 13 weeks. The remaining 96% of tumors remain microscopic, non-angiogenic, and dormant due to their inability to recruit new blood vessels [29]. In another study, severe combined immunodeficient (SCID) mice were inoculated with non-angiogenic human MDA-MB-436 breast adenocarcinoma cells, KHOS-24OS osteosarcoma cells, or T98G glioblastoma cells [29, 30]. While most resulting tumors remained microscopic, with a diameter of less than 1 mm, some eventually turned angiogenic and enlarged sufficiently for isolation of angiogenic tumor cells. These cells were subsequently inoculated into SCID mice alongside non-angiogenic counterparts to determine the time until palpable tumor development. Researchers assessed in vitro cell proliferation via growth curves and evaluated in vivo proliferation through proliferating cell nuclear antigen or Ki-67 staining techniques. Fourteen days post-inoculation, they histologically examined tumors from both cell populations for vascular development while analyzing thrombospondin-1 (TSP-1) expression using immunoblotting methods. Non-angiogenic tumors required an average period of 119 d (range 53–185 d) for breast cancer, 238 d (184–291 d) for osteosarcoma, and 226 d (150–301 d) for glioblastoma before developing palpable tumors. Conversely, angiogenic cells developed palpable tumors within just 20 d. Despite exhibiting similar proliferation rates in vitro, angiogenic tumors displayed functional vasculature after 14 d, while non-angiogenic counterparts remained microscopic with absent or dysfunctional vasculature. Notably, angiogenic cells showed significantly lower TSP-1 expression, ranging from 5- to 23-fold lower depending on tumor type, compared with non-angiogenic counterparts. This model provides a robust in vivo system along with a conceptual framework for exploring the initiation mechanisms underlying the reversibility and molecular regulation of the angiogenic switch, addressing essential questions in cancer biology research [29].

Angiogenic dormancy emerges when there is an imbalance between pro-angiogenic and anti-angiogenic factors, leading to the predominance of anti-angiogenic elements. Consequently, the formation of new blood vessels is inhibited. Key molecules contributing to this anti-angiogenic state include TSP-1, angiostatin, and endostatin. These molecules function by inhibiting the proliferation and migration of endothelial cells, which are vital for blood vessel development [31]. An experimental study employing a xenograft model in SCID mice with U-87 human glioblastoma cells verified this mechanism [32]. The study revealed that the “dormant” clones of U-87 cells formed smaller tumor masses without blood vessels, while the “aggressive” clones developed larger vascularized tumors. Interestingly, the dormant clones exhibited elevated levels of TSP-1, a molecule known for inhibiting angiogenesis and impeding tumor invasion. These findings suggest that high expression levels of TSP-1 in dormant clones contribute to the suppression of angiogenesis and the slower progression of tumor growth. This illustrates how elevated TSP-1 levels can shift the balance towards anti-angiogenic factors, thereby preventing the “angiogenic switch” and maintaining tumors in a dormant and non-vascularized state [33, 34].

The TME also plays a pivotal role in maintaining angiogenic dormancy. A common feature of dormant tumors is hypoxia or low oxygen levels. This condition facilitates the stability of hypoxia-inducible factors (HIFs), which are transcription factors responsive to reduced oxygen levels in the cellular environment [35, 36]. HIFs can induce the expression of both pro-angiogenic factors such as vascular endothelial growth factor (VEGF), and anti-angiogenic factors like TSP-1. The balance between these opposing forces determines whether a tumor remains dormant or transitions to an angiogenic state and begins to proliferate [37, 38]. In a hypoxic environment, the stabilization of HIFs may lead to the upregulation of VEGF, thereby promoting angiogenesis and potentially shifting the balance toward tumor growth. Conversely, the expression of anti-angiogenic factors can mitigate this effect and help sustain angiogenic dormancy. This interplay among various factors is complex, and the precise mechanisms are still being actively explored. Changes within the TME can trigger the transition from angiogenic dormancy to active proliferation. Factors such as inflammation, genetic mutations, or alterations in the balance between pro- and anti-angiogenic factors can stimulate angiogenesis. Once initiated, angiogenesis enables tumors to grow rapidly and become more aggressive.

Understanding angiogenic dormancy is essential for the development of novel cancer therapies. Anti-angiogenic treatments aim to maintain tumors in a dormant state by inhibiting blood vessel formation. Agents such as bevacizumab, which specifically target and inhibit the process of angiogenesis, have been utilized in various cancer types. However, tumors can acquire resistance to these therapies by activating alternative pathways for vascular formation and survival. This adaptive response encompasses the utilization of VEGF-independent angiogenic factors, such as fibroblast growth factor (FGF) and platelet-derived growth factor [39, 40], upregulation of HIFs to stimulate diverse pro-angiogenic pathways [41], and engagement with the angiopoietin-Tie2 system [27, 41]. Additionally, tumors may exploit paracrine signaling mechanisms by secreting cytokines that activate adjacent stromal cells to promote angiogenesis [27, 40]. Changes in the TME, including the recruitment of pro-angiogenic cells such as macrophages and endothelial progenitor cells, further exacerbate this resistance [39, 40]. These multi-faceted strategies enable tumors to maintain their blood supply and continue proliferating despite targeted anti-angiogenic interventions, highlighting the necessity for combination treatments that simultaneously address multiple pathways. Ongoing research aims to identify new targets and strategies for maintaining or inducing angiogenic dormancy. The integration of anti-angiogenic therapies with other modalities, such as immunotherapy or targeted therapy, may improve their efficacy. Such approaches hold promise for enhancing long-term cancer control by preventing the switch from dormancy to active growth.

### Immunological dormancy

Immunological dormancy pertains to a condition wherein the immune system effectively governs and restrains tumor cells, thereby preventing their proliferation and spread. In this condition, the immune system identifies and suppresses cancer cells, maintaining them in a dormant and non-proliferative state. The immune system plays a crucial role in surveilling and eliminating malignant cells. The key components of this process encompass cytotoxic T lymphocytes (CTLs), natural killer (NK) cells, and diverse cytokines. CTLs and NK cells are capable of recognizing and killing cancer cells, while cytokines such as interferons function to modulate the immune response and inhibit tumor growth [42, 43].

Tumor cells can enter a state of immunologic dormancy when the immune system proficiently recognizes and targets them. This recognition is frequently facilitated by tumor antigens present on the surfaces of cancer cells. These antigens are identified by immune cells, triggering an immune response that keeps the tumor under control. Tumor immune surveillance conducted by the host’s immune system is a major element in establishing and maintaining tumor dormancy. The immune system, particularly T cells and NK cells, can recognize tumor antigens and render proliferating tumor cells functionally dormant through cytotoxic activity [44, 45]. The expression of major histocompatibility complex class I molecules on tumor cells presents these antigens to T cells, potentially prolonging the dormant state by enabling effective immune recognition and elimination of the tumor cells. Loss of major histocompatibility complex class I molecules expression may result in immune evasion and escape from dormancy [46]. During the equilibrium phase of cancer immunoediting, the immune system maintains a functional balance with tumor cells to keep them dormant. This process is supported by various immune factors such as T cells, NK cells, interferon-γ (IFN-γ), and IL-12 recognizing tumor antigens while exerting anti-tumor effects [45, 47].

In recent years, chimeric antigen receptor (CAR) T cells have emerged as a promising approach in cancer immunotherapy, presenting a novel perspective for managing the dormant state of cancer cells. CAR T cells are genetically engineered T lymphocytes that are designed to target and eliminate cancer cells with high specificity [48, 49]. By incorporating an antigen-binding domain derived from a monoclonal antibody along with T-cell activation domains, CAR T cells can precisely recognize tumor-associated antigens and elicit a robust immune response. This targeted approach not only enhances the destruction of actively proliferating cancer cells but also has the potential to influence the state of immunogenic dormancy. However, the role of CAR T cells in inducing and maintaining tumor dormancy remains inadequately defined. By effectively targeting and eliminating the actively dividing cancer cells, CAR T cells can reduce the overall tumor burden, thereby creating a microenvironment conducive to sustaining any residual cancer cells in a dormant state [50]. This strategic reduction in tumor load aids in controlling cancer progression while minimizing the risk of metastatic spread. Additionally, CAR T cells provide a continuous surveillance mechanism capable of detecting and responding to any signs of cancer cell reactivation. Once engineered for persistence within the patient’s body, these cells are prepared to recognize and promptly attack any reactivated cancer cells, thus preventing relapse and ensuring disease control over time [51, 52]. Furthermore, CAR T cells can modulate the TME, making it less favorable for the activation of dormant cancer cells. Through the secretion of cytokines and chemokines, CAR T cells recruit and activate other immune components, amplifying the overall anti-tumor response while reinforcing the state of dormancy [53, 54]. Several challenges need to be addressed to fully exploit the potential of CAR T cells in managing immunogenic dormancy. Concerns such as antigen escape, where tumor cells downregulate or lose targeted antigens, along with the risks of off-target effects and cytokine release syndrome, remain significant obstacles. Moreover, ensuring the long-term persistence and functional effectiveness of CAR T cells is crucial for sustained control over dormant cancer cells.

One of the mechanisms involved in immunologic dormancy is known as immune editing. This process consists of three phases: elimination, equilibrium, and escape. During the elimination phase, both innate and adaptive immune responses collaborate to identify and eliminate cancerous cells. The innate immune system, which includes components such as NK cells and complement proteins, plays a vital role in this initial immune response. These elements work together to detect and destroy transformed cells, thereby preventing further proliferation [55, 56]. This process is facilitated by signals produced by the tumor that are associated with its presence. These signals can trigger acute inflammatory responses, promoting the recognition and destruction of tumor cells by various immune cells, including NK cells, dendritic cells, macrophages, and tumor-specific T cells, which are essential for inhibiting tumor cells while contributing to the phenomenon of immune editing [57]. If the elimination phase fails to achieve complete success, any remaining tumor cells enter the equilibrium phase. In this phase, the immune system exerts control over the growth of tumor while maintaining these tumor cells in a functionally dormant state. This equilibrium state represents a delicate balance between the ability of immune system to suppress tumor growth and the capacity of tumor cells to evade complete eradication [55, 57]. It reflects a dynamic interaction between anti-tumor factors (e.g., IL-12, IFN-γ) and pro-tumor factors (e.g., IL-10, IL-23). The adaptive immune system, particularly T cells, is crucial for maintaining this state of dormancy [56]. However, the persistent immunological pressure during the equilibrium phase may lead to the selection for variant tumor cell populations that acquire mutations, enabling them to evade immunity. Such adaptations include the loss of tumor antigens, defects in antigen presentation, and the induction of immunosuppression via programmed death-ligand 1 (PD-L1) [56, 57]. Upon the emergence of such variants, they transition into the escape phase, becoming clinically evident and establishing an immunosuppressive TME that facilitates progressive tumor growth and relapse [55, 56].

Immunologic dormancy is a highly dynamic process that can be disrupted by various factors. Changes in the TME, alterations in immune cell functionality, or the acquisition of immune-evasive mutations by tumor cells can disrupt the balance, leading to the tumor reactivation. Additionally, stress, infections, or other systemic changes may adversely affect immune surveillance and contribute to the exit from dormancy [58].

Understanding immunologic dormancy is of crucial significance for advancing immunotherapy that either maintains this dormant state or reactivates the immune system to eliminate residual cancer cells. Immune checkpoint inhibitors, such as programmed death-1 (PD-1)/PD-L1 or cytotoxic T-lymphocyte-associated protein 4 (CTLA-4), hold great promise in reawakening the immune response against tumors [59, 60]. These therapies enhance immune surveillance by blocking inhibitory signals that prevent immune cells from attacking cancer cells. Furthermore, researchers are investigating cancer vaccines and adoptive cell transfer therapies to strengthen the immune system’s ability to recognize and target dormant tumor cells [61, 62]. These strategies aim to improve the presentation of tumor antigens and activate immune cells, thereby supporting the maintenance of immunologic dormancy or facilitating the eradication of dormant cancer cells.

## Mechanisms underlying tumor dormancy

### Genetic and epigenetic factors

Genetic and epigenetic factors play a pivotal role in the mechanisms underlying tumor dormancy. These factors influence the ability of cancer cells to enter, maintain, and exit a dormant state [63, 64]. Genetic alterations in the DNA sequence of cancer cells include mutations, deletions, amplifications, and translocations. Mutations in oncogenes and tumor suppressor genes are particularly significant. When mutated, oncogenes can drive continuous cell division. Conversely, inactivated tumor suppressor genes fail to regulate the cell cycle effectively [65, 66]. For example, mutations in oncogenes, such as phosphatidylinositol-4,5-bisphosphate 3-kinase catalytic subunit alpha (*PIK3CA)* occur in approximately 12% of patients with gastric cancer [67], kirsten rat sarcoma virus (*KRAS)* is mutated in about 20.4% of non-small cell lung cancer patients [68], while B-Raf proto-oncogene, serine/threonine kinase (*BRAF)* mutations are found in around 10% of colon cancer [69] and 2–4% of non-small cell lung cancer patients [70, 71]. In dormant cells, genetic changes may facilitate survival under stress but prevent active proliferation [65, 72]. For example, mutations in the *TP53* gene, responsible for coding the p53 protein, can promote cell survival despite DNA damage. The p53 protein typically induces cell cycle arrest or apoptosis following damage. However, mutant Protein 53 (*p53*) enables cells to survive in a quiescent state, contributing to dormancy [73]. Similarly, mutations in the phosphatase and tensin homolog (*PTEN*) gene can activate the PI3K/Akt pathway, enhancing survival signals while preserving dormancy [73, 74].

Epigenetic alterations are modifications that affect gene activity without changing the DNA sequence. These changes encompass DNA methylation, histone modification, and the regulation of non-coding RNAs. Specifically, DNA methylation involves the addition of methyl groups to the DNA molecule, which typically results in the suppression of gene expression. Histone modifications, such as acetylation and methylation, affect how tightly DNA is wound around histone proteins, thereby influencing gene accessibility [75]. In dormant cancer cells, epigenetic changes can silence genes required for cell cycle progression. For instance, hypermethylation of promoters for cell cycle-related genes like *cyclin D2* can inhibit cell division [76, 77]. Additionally, histone deacetylations (HDACs) can compact chromatin structure, making genes less accessible for transcription [78]. HDACs serve a regulatory function in various physiological processes that contribute to cancer progression and metastasis. Through deacetylating numerous substrates and interacting with various proteins, HDACs influence critical cellular functions including cell growth, programmed cell death, cell motility, the transition from epithelial to mesenchymal states, and the formation of new blood vessels [79]. Recent studies have highlighted the role of HDAC inhibitors (HDACi) in tumor dormancy [80, 81]. For instance, HDACi are being investigated for their potential to induce dormancy in cancer cells by modulating the acetylation of histone and non-histone proteins. This modulation affects genes that regulate the cell cycle, apoptosis, and differentiation. Research has demonstrated that HDACi can increase the expression of the leukemia inhibitory factor receptor, a critical regulator of dormancy. In breast cancer, elevated leukemia inhibitory factor receptor levels are associated with reduced cell proliferation and improved survival outcomes. Clinical trials indicate that HDACi can promote pro-dormancy gene expression, potentially leading to prolonged survival in patients with metastatic breast cancer [80].

Non-coding RNAs, encompassing microRNAs (miRNAs) and long non-coding RNAs (lncRNAs), are involved in tumor dormancy and post-transcriptional regulation of gene expression [82]. miRNAs play a pivotal role in regulating tumor dormancy by targeting key cellular mechanisms that influence tumor cell proliferation, metastasis, and immune response. For instance, the miR-200 family targets mesenchymal transcription factors, thereby reducing proliferation and promoting a dormant state in breast and bone cancers [83, 84]. Similarly, miR-335 suppresses SRY-box transcription factor 4 expression, inhibiting metastatic growth in breast cancer, and contributing to dormancy [85]. miR-190 induces quiescence by targeting angiogenesis and antigenic genes, particularly in bone cancer and glioblastoma [86]. Conversely, miR-222, miR-127, miR-197, and miR-223 target C-X-C motif chemokine ligand 12 (*CXCL12)* and other associated genes, decreasing proliferation and impairing the tumor immune response in lung cancer, further enhancing dormancy [87]. Additionally, miR-221 and miR-222 target p27, a key regulator of cell cycle progression, leading to tumor cell cycle arrest and dormancy in leukemia [88]. These miRNAs underscore the complex regulatory network that governs tumor dormancy and highlight their potential as therapeutic targets for controlling metastasis and relapse. Furthermore, miRNAs can bind to messenger RNAs, inhibiting their translation into proteins or inducing their degradation. Specific miRNAs in dormant cells can downregulate genes associated with cell proliferation [89]. For example, miR-34a targets and reduces the expression of E2F3, a transcription factor essential for cell cycle progression [90, 91]. In human papillomavirus-positive cervical cancer cells, miR-34a directly interacts with E2F3. Through this interaction, miR-34a regulates survivin levels. Consequently, by modulating both E2F3 and survivin, miR-34a has the potential to diminish the survival and invasive capabilities of these cancer cells [91].

Moreover, lncRNAs contribute to tumor dormancy through diverse mechanisms that regulate cellular quiescence, metastasis, and cancer stem cell (CSC) phenotypes. For example, NRF2F1-AS1 promotes cellular quiescence in breast cancer via transcriptional regulation, thereby helping cells maintain a dormant state [92]. Similarly, MALAT1 influences transcription and alternative splicing, leading to reduced proliferation and metastasis in breast cancer, thus enhancing dormancy [93]. The lncRNA FOXF1-AS1 interacts with EZH2 to modulate CSC phenotypes in lung cancer, contributing to tumor dormancy by maintaining the stem-like properties of cancer cells [94]. Additionally, HAL and NEAT1 regulate chromatin remodeling and influence CSC phenotypes in breast and colorectal cancers, further promoting a dormant state [95]. LncRNAs such as ARHGAP5-AS1 stabilize the ARHGAP protein, leading to chemoresistance in gastric cancer and indirectly influencing dormancy by enabling cancer cells to survive in a dormant, drug-resistant state [96]. H19, packaged in exosomes and delivered to the TME, promotes angiogenesis in liver cancer, thereby maintaining a non-proliferative, dormant-like state in the tumor [97]. LncRNAs like HOTAIR interact with the polycomb repressive complex 2 to mediate chromatin structure, influencing proliferation, CSC phenotypes, and chemoresistance in multiple cancers [98, 99]. Lastly, BORG interacts with tripartite motif-containing 28 and replication protein A1, regulating proliferation, CSC phenotypes, and chemoresistance in breast cancer, which may also play a role in maintaining tumor dormancy [100, 101]. These lncRNAs are crucial for regulating the balance between dormancy, survival, and metastasis, making them important targets for therapeutic strategies.

Epigenetic plasticity refers to the ability of an organism’s epigenome to adapt and undergo changes in response to environmental factors, life experiences, and developmental stages. This plasticity enables dormant cells to adjust to environmental fluctuations, allowing them to reversibly switch between active and inactive states based on external signals [63]. A key feature of epigenetic plasticity in cancer cells is the regulation of gene expression through alterations in chromatin structure, histone modifications, DNA methylation, and non-coding RNAs. These epigenetic changes can activate or silence specific genes without altering the underlying DNA sequence, enabling cancer cells to dynamically adapt their phenotypes. HIFs are central regulators of cellular responses to low oxygen conditions (hypoxia), a common characteristic of the TME [102, 103]. Under hypoxic conditions, HIFs stimulate the expression of genes that promote survival by activating pathways involved in angiogenesis [104, 105], metabolic adaptation [106, 107], and stress resistance [108]. Simultaneously, HIFs inhibit genes associated with cell cycle progression, thereby limiting proliferation and inducing a quiescent state [109, 110]. This dynamic shift between survival and dormancy is essential for cancer cells to withstand fluctuating microenvironmental conditions and evade premature exhaustion or immune attack. For instance, under low oxygen conditions, HIFs can stimulate the expression of genes that promote survival while inhibiting proliferation [111, 112].

Furthermore, genetic and epigenetic changes interact with each other. Genetic mutations can lead to epigenetic changes and vice versa [113, 114]. For example, mutations in *IDH1* and *IDH2* genes produce an oncometabolite known as 2-hydroxyglutarate (2-HG) [115]. These genetic alterations confer a novel enzymatic function to the affected enzymes, enabling them to convert α-ketoglutarate into the cancer-associated metabolite 2-HG [116, 117]. The oncometabolite 2-HG tends to accumulate in various cancers. Elevated levels of 2-HG can suppress the activity of the ten-eleven translocation family of DNA demethylases as well as the Jumonji family of histone demethylases. This inhibition may result in alterations in chromatin architecture and gene expression that contribute to tumor development, leading to widespread epigenetic modifications that promote dormancy [118, 119].

The interplay between genetic and epigenetic factors is complex and contributes to the heterogeneity observed in dormant cells. This heterogeneity poses significant challenges for targeting dormant cells with conventional therapies [6, 11]. A comprehensive understanding of the specific genetic and epigenetic landscape of dormant cells is essential for the development of targeted therapeutic strategies.

### Microenvironmental influences

Microenvironmental influences play a pivotal role in the mechanisms underlying tumor dormancy. The TME is a complex and dynamic entity composed of various cellular and non-cellular components interacting with cancer cells [120]. These interactions are of paramount importance for maintaining the dormant state of tumor cells and can significantly affect their behavior and fate [121].

#### Role of ECM

The ECM is an intricate network of proteins, glycoproteins, proteoglycans, and other molecules that provide structural and biochemical support to the cells within tissues. It acts as a dynamic element of the tissue microenvironment and plays a vital role in regulating various cellular functions. The ECM comprises proteins such as collagen, fibronectin, and laminin [122, 123]. Dormant tumor cells often reside in niches where the composition of ECM promotes a non-proliferative state [123]. Integrins, which are cell surface receptors, mediate the interactions between cells and the ECM. These interactions can activate signaling pathways that induce cell cycle arrest and dormancy [19]. For example, the binding of integrins to ECM components like fibronectin can activate focal adhesion kinase (FAK) along with downstream signaling pathways that support dormancy [124]. Alterations in the ECM, such as changes in stiffness or composition, can trigger dormant cells to exit their dormancy and resume proliferation [122, 123]. A recent study reported that disseminated tumor cells (DTCs) maintain dormancy by remodeling their ECM to create a niche enriched with type III collagen, which is crucial for sustaining this state. Disruption of this collagen network activates tumor proliferation via DDR1-STAT1 signaling pathway. Advanced imaging techniques revealed that exiting dormancy correlates with alterations in the levels of type III collagen. Clinical samples confirm elevated levels of type III collagen in lymph node-negative head and neck squamous cell carcinoma (HNSCC) patients [125].

DTCs and circulating tumor cells (CTCs) are of crucial significance in cancer metastasis, but they differ in terms of location, physiological state, and the role they play in disease progression. DTCs detach from primary tumors and spread to distant organs, where they may remain dormant or in a slow-cycling state for extended periods. This dormancy makes them difficult to detect and treat, posing a substantial challenge as they can lead to metastatic relapses long after the primary tumor has been treated [126].

The dynamics of CTCs and DTCs are crucial for understanding tumor dormancy and metastasis. CTCs, which are shed from primary tumors into the bloodstream, serve as markers of early-stage metastasis and are generally more active than DTCs, which may remain dormant for extended periods at distant sites. Dormant DTCs can enter a quiescent state, evading immune detection and therapeutic interventions while retaining the potential to reactivate and proliferate, leading to late recurrences of aggressive disease [127, 128]. Studying cancer cell dormancy is challenging due to the difficulties in acquiring invasive samples, particularly from bone marrow, making the assessment of biomarkers on CTCs a promising alternative. For example, Ki-67 and M30 are markers for proliferation and apoptosis, respectively [129, 130]. These markers were evaluated in CTCs from early-stage and metastatic breast cancer patients, revealing that 82.4% of CTCs in early-stage patients were dormant (Ki-67^−^/M30^−^), compared with 59.1% in metastatic patients [127, 131]. This suggests that monitoring CTCs for dormancy and apoptosis could help predict disease recurrence. Further research identified urokinase plasminogen activator receptor/integrin subunit beta 1 as markers of dormancy in epithelial cell adhesion molecule negative CTCs, which possess stem cell properties promoting brain metastasis, and showed that these CTCs had elevated expression of genes related to DNA repair and blood–brain barrier permeability. Additionally, mTOR complex 2 (mTORC2) activation and reduced mTORC1 signaling were linked to the long-term dormancy of DTCs in bone marrow [132, 133]. These findings highlight the potential of CTC biomarkers in elucidating tumor dormancy mechanisms and advancing personalized cancer treatment strategies.

#### Impact of hypoxia

Hypoxia, or low oxygen levels, is a prevalent characteristic of the TME that affects tumor dormancy [134]. Under hypoxic conditions, cells activate HIFs, which serve as master regulators of immune escape in tumors [36]. The activation of HIF-1α triggers a cascade of signaling events [135]. Additionally, hypoxia promotes the release of complex class I chain-associated molecules by impairing nitric oxide signaling, thereby disrupting immune surveillance conducted by NK cells [36]. HIF-1α, a key regulator in hypoxic environments, can induce a metabolic shift towards glycolysis, enabling cells to survive with reduced oxygen availability [136]. This adaptation not only supports cell viability in a dormant state but also prepares cells for rapid proliferation if environmental conditions improve.

#### Immune system regulation

Studies have indicated that the immune system can regulate the tumor dormancy [58, 137]. The immune system is a complex network of cells, tissues, and organs that collaborate to protect the body against infections, diseases, and foreign substances. It plays a dual role in tumor dormancy. On one hand, immune surveillance can maintain dormancy by recognizing and eliminating proliferating tumor cells. On the other hand, immune cells and their secreted factors can create an immunosuppressive microenvironment that supports this dormant state [45, 138]. For example, CTLs [139] and NK cells [140], target active tumor cells to help maintain a dormant population. Moreover, immune-modulating cytokines such as IFN-γ can induce dormancy by promoting cell cycle arrest [141]. IFN-γ functions alongside granzyme B and perforin as a cytotoxic cytokine to induce apoptosis in tumor cells [142]. However, chronic exposure to IFN-γ may facilitate the development of certain cancer types. Furthermore, chronic inflammation coupled with the presence of immunosuppressive cells like regulatory T cells (Tregs) and myeloid-derived suppressor cells (MDSCs) can disrupt the balance, leading to reactivation of tumor cells.

#### Role of stromal cells

Stromal cells also play a crucial role in tumor dormancy. These supportive cells are found in the connective tissue of organs, providing structural and biochemical support to the parenchymal cells of the tissue. Stromal cells, including fibroblasts, endothelial cells, and mesenchymal stem cells (MSCs), constitute critical components of the TME. Cancer-associated fibroblasts (CAFs) are particularly pivotal in the TME, exhibiting heterogeneity and significantly contributing to tumor growth, angiogenesis, invasion, and metastasis, as well as ECM remodeling and chemoresistance. Their interaction with tumor cells is essential for tumorigenesis and progression. Moreover, CAFs influence the TME by secreting a variety of molecules, including cytokines, growth factors, and chemokines, which collectively create an immunosuppressive environment facilitating cancer cell evasion from immune surveillance, thereby underscoring their multi-faceted role in cancer progression [143]. One of the key factors secreted by CAFs is transforming growth factor-β (TGF-β), whose signaling exhibits unique characteristics during cancer progression and presents potential targets for anti-cancer therapy [144]. Members of the TGF-β family can influence nearby cells like CAFs to restrain tumor growth and spread during the initial phases of disease. Nevertheless, as the disease progresses to more advanced stages, TGF-β signaling shifts towards promoting tumor growth [145]. In the context of tumor dormancy, TGF-β has been shown to induce dormancy under specific conditions by activating signaling pathways that inhibit cellular proliferation. This is supported by evidence indicating that fibroblasts losing their responsiveness to TGF-β lead to abnormal production of growth factors and cytokines. Such alternations in fibroblast behavior are associated with the development of premalignant and malignant lesions in epithelial tissues, such as those of stomach, prostate, and breast [146].

#### Endothelial cells and angiogenesis

Endothelial cells, which are pivotal in angiogenesis, play a crucial role in the formation of new blood vessels, an essential process for embryonic development, adult tissue maintenance, and tumor progression. In the context of cancer, these cells are significant in disrupting the dormancy of tumor cells that reside within avascular niches, where the balance between proliferation and apoptosis is maintained due to inadequate vascularization, limiting tumor growth to a microscopic, asymptomatic, and non-metastatic state [147, 148]. The angiogenic switch represents a critical mechanism whereby dormant cells stimulate the formation of new blood vessels, facilitating their exit from dormancy and subsequent tumor expansion. This transition is vital for the rapid growth and enlargement of tumor masses as well as for initiating metastatic process. It predominantly relies on a shift in equilibrium within the TME, transitioning from anti-angiogenic to pro-angiogenic factors that favor the latter [148]. VEGF is central to both angiogenesis and vasculogenesis, the processes responsible for expanding existing blood vessels and generating new ones. It is also essential for embryonic development, vascular repair mechanisms, and tumor proliferation. During embryonic stages, VEGF levels are significantly elevated and works synergistically with various endothelial growth factors to regulate neovascularization [149]. Solid tumors also exploit VEGF to bolster their neoplastic growth. The upregulation promotes enhanced vascularization within tumors, thereby facilitating malignant progression [149]. Under hypoxic conditions, tumor cells secrete various growth factors that contribute to angiogenesis. Among these factors, VEGF hold particular significance as they activate VEGF receptor-2, a key mediator that promotes angiogenesis while alleviating oxygen deprivation within the central region of tumors [150]. However, in the context of tumor dormancy, the role of VEGF becomes more complex. Dormant tumor cells, particularly DTCs residing in distant organs such as bone marrow, often enter a quiescent state, evading immune detection and therapy [13, 128]. Despite their dormant state, these cells retain the potential to reactivate and proliferate, often influenced by changes in the TME, including fluctuations in VEGF levels. The upregulation of VEGF can trigger angiogenesis, reawakening dormant cells by increasing blood supply and providing the necessary nutrients and oxygen for their growth. Consequently, while VEGF’s role in promoting angiogenesis is well established in active tumor growth, it also plays a pivotal role in transitioning dormant tumors back to a proliferative state, contributing to late recurrences and metastasis after prolonged dormancy [124, 151].

#### Mechanical forces

Mechanical forces significantly influence tumor dormancy. In the context of tumors, mechanical forces refer to the physical stresses exerted within and around tumor tissues, including compression, tension, and shear stress. These forces emerge from tumor growth, interactions with the surrounding ECM, and fluid pressure within the TME. They can modulate cancer cell behavior, affect tumor progression and metastasis, as well as impact the efficacy of therapeutic interventions. Mechanical forces within TME, encompassing tissue stiffness and interstitial fluid pressure, play a vital role in regulating cellular dormancy [152]. Increased tissue stiffness, often resulting from ECM remodeling processes, can activate integrin signaling pathways that promote a dormant state [153]. The ECM is integral to cancer progression and dormancy, influencing the characteristics of tumor cells and their metastatic potential. The remodeling and degradation of the ECM are essential processes that facilitate the initial stages of metastasis, including invasion into adjacent tissues, intravasation into the bloodstream by tumor cells, and subsequent extravasation into new tissues [154]. For example, the composition of the ECM can create a microenvironment that supports tumor cell dormancy. Specifically, a dense and fibrotic ECM can limit the availability of growth factors and nutrients, effectively “sheltering” dormant tumor cells from proliferative and invasive signals. This protective environment helps maintain the quiescent state of these cells, preventing them from entering the cell cycle [155, 156].

Integrins, transmembrane receptors with unique ligand-binding capabilities on their extracellular domains, also possess signal transduction functions within their cytoplasmic regions. The signaling pathways activated by integrins, including those involving Ras- and Rho-GTPase, TGF-β, Hippo, Wnt, Notch, and Sonic Hedgehog, are implicated at various stages in cancer development. For example, research has shown that the absence of β1 integrin in breast cancer models activates the tumor suppressor gene p53, leading to cellular senescence and dormancy. Tumors deficient in β1 integrin exhibited dormancy traits, such as reduced proliferation and increased apoptosis, and when p53 was mutated or deleted, these tumors bypassed dormancy. This suggests that β1 integrin mediates p53-dependent responses that promote dormancy [19, 157]. Additionally, β1 integrin signaling regulates cancer cell dormancy through its downstream molecule, FAK. Inhibiting β1 integrin signaling with antibodies induced growth arrest in mammary cancer cells, highlighting the pivotal role of FAK signaling in maintaining dormancy [19, 158]. Therefore, a comprehensive understanding of these intricate regulatory mechanisms and molecular distinctions associated with integrins is vital for impeding cancer progression and preventing tumorigenesis [153]. Conversely, changes in mechanical forces may disrupt these signals, leading to re-entry into the cell cycle [152]. Mechanical stress has been shown to trigger re-initiation of the cell cycle along with transition into the S phase. Yes-associated protein 1 remains localized in the cytoplasm and cortex under conditions without mechanical stress, while β-catenin is found at cell–cell junctions. However, both proteins relocated to the nucleus upon exposure to strain despite at varying rates [159].

#### Nutrient availability

The availability of nutrients in the TME also plays a critical role in regulating tumor dormancy. Dormant tumor cells often reside in regions deprived of nutrients, compelling them to adapt their metabolism [23]. One notable adaptation is autophagy, a catabolic process through which cells degrade and recycle their components. Autophagy is essential for the adaptation, survival, and reactivation of dormant cells [160]. It is activated under adverse conditions and promotes cellular maintenance as well as survival while also facilitating cell death. Therefore, understanding the role of autophagy in regulating the functions of cancer cells, including those of dormant cells, is of great interest [160, 161]. Fluctuations in nutrient levels can signal dormant cells to either remain quiescent or reactivate and proliferate. The metabolic adaptations observed in dormant tumor cells are influenced by factors such as tissue origin characteristics, environmental nutrient availability, and stromal influence [23]. A reduced metabolic rate coupled with an increased resistance to oxidative stress often characterizes these dormant cells. However, various elements such as the composition of the ECM, stromal cell influence, and nutrient accessibility can induce distinct alterations in these dormant cells, which may ultimately lead to tumor resurgence [23].

## Mechanisms of tumor relapse

### Reactivation of dormant cells

The reactivation of dormant cancer cells represents a critical mechanism underlying tumor relapse. Dormant cells, which have remained in a non-proliferative state, can resume proliferation and contribute to disease recurrence. Various factors influence this reactivation, involving complex biological processes.

A pivotal factor in the reactivation of dormant cancer cells is the alteration of TME, which provides signals that can either sustain dormancy or stimulate cell proliferation. For example, recent research using mouse models has uncovered how persistent lung inflammation induced by tobacco smoke or lipopolysaccharide awakens dormant cancer cells, leading to the formation of metastases [162]. This process involves the generation of neutrophil extracellular traps (NETs), which are essential for reactivating dormant cells. Two NET-associated proteases, neutrophil elastase and matrix metalloproteinase 9, remodel laminin, thereby activating integrin α3β1 signaling and prompting the proliferation of dormant cells. Antibodies targeting remodeled laminin have been shown to inhibit this awakening, indicating that modulating this pathway could enhance the survival of cancer patients by preventing metastasis [163]. Furthermore, growth factors and cytokines play a vital role in reactivation. These molecules are frequently produced in response to tissue injury or inflammation. For instance, TGF-β and IL-6 are recognized for their ability to stimulate cancer cell proliferation. When dormant cells encounter these growth factors, they may be induced to exit dormancy and resume cellular division [164]. Moreover, hypoxia within the TME can significantly affect both dormancy and reactivation of cancer cells. HIFs are stabilized under low oxygen conditions and can elicit a range of cellular responses. Initially, hypoxia may contribute to the maintenance of dormancy by restricting energy resources [18]. However, if the hypoxic environment undergoes changes, such as through angiogenesis that restores oxygen supply, dormant cells have the potential to be reactivated.

Another significant mechanism involves the immune system. Dormant cells can evade immune detection by downregulating the expression of tumor antigens or by establishing an immunosuppressive microenvironment [19, 165]. However, changes in immune surveillance may lead to reactivation. For example, a decline in immune function due to aging, stress, chronic inflammation, or immunosuppressive treatments can reduce the control over dormant cells, facilitating their proliferation.

### Genetic and epigenetic changes

The mechanisms underlying tumor relapse often involve genetic and epigenetic changes that drive cancer cells to escape dormancy and reinitiate growth. Genetic modifications, such as mutations, amplifications, deletions, and chromosomal rearrangements, can occur in key genes associated with tumor suppression, cell cycle regulation, and DNA repair pathways. These alterations may confer growth advantages to cancer cells and facilitate their survival under adverse conditions.

Mutations in tumor suppressor genes, such as *TP53* [166], *PTEN* [167], and *RB1* [168], can disrupt cell cycle checkpoints and apoptosis pathways, enabling cancer cells to evade growth control mechanisms. Additionally, mutations in oncogenes, such as *KRAS* [169], *BRAF* [170], and human epidermal growth factor receptor 2 (*HER2)* [171], can drive aberrant cell proliferation and survival signaling, thereby contributing to tumor relapse. Amplification or overexpression of proto-oncogenes, such as *MYC* [172] and *cyclin D1* [173], can also promote tumor growth and relapse by facilitating cell cycle progression and genomic instability. Epigenetic changes, including DNA methylation [174], histone modifications [175], and non-coding RNA regulation [176], also play a critical role in tumor relapse. Disrupted DNA methylation, characterized by increased methylation at tumor suppressor genes coupled with decreased methylation at oncogenes, may lead to the silencing of protective genes while activating detrimental ones, thus promoting tumor growth and recurrence [177]. Similarly, changes in histone acetylation and methylation can reshape chromatin structure and gene activity, affecting the behavior and evolution of tumor cells [178].

Non-coding RNAs, such as miRNAs and lncRNAs, modulate gene expression post-transcriptionally, thereby impacting tumor relapse. They are essential for cancer dormancy and recurrence, with miRNAs influencing cell cycle regulation and growth signaling, while specific miRNA profiles can predict relapse in triple-negative breast cells [179, 180]. Similarly, lncRNAs exhibit both oncogenic and tumor-suppressive properties that impact cancer cell proliferation, migration, invasion, and prognosis. They also affect macrophage differentiation, which is a key factor for immune evasion [181]. Moreover, epigenetic changes within the TME further contribute to tumor relapse by creating an immunosuppressive environment that impedes the efficacy of immunotherapy [182]. Additionally, hypoxia, an inherent characteristic of solid tumors due to rapid growth outpacing oxygen supply, is associated with increased chemoresistance, radioresistance, disease relapse, and metastasis [134, 183]. The hypoxic TME not only contributes to aggressive tumor behavior but also promotes tumor dormancy by inducing metabolic adaptations that enable cancer cells to survive in low-oxygen conditions. This dormant state can persist for extended periods, evading immune surveillance and conventional therapies. Tumor cells in this dormant state may remain phenotypically stable, yet they harbor the potential for reactivation, particularly when favorable conditions arise, such as altered signaling within the TME or the reoxygenation of previously hypoxic areas. Reactivation often leads to rapid proliferation, metastasis, and resistance to standard treatment protocols, complicating long-term cancer management and contributing to relapse [18, 184]. Inflammation is another hallmark of cancer linked to metastatic recurrence and resistance to treatment [185, 186]. Inflammatory cytokines and immune cells within the TME can create a microenvironment conducive to both tumor dormancy and reactivation. Chronic inflammation may promote the survival of dormant cancer cells by enhancing immune evasion mechanisms, such as the recruitment of immunosuppressive Tregs and the secretion of anti-apoptotic factors. However, inflammation can also trigger the reawakening of dormant tumor cells through the production of growth factors and cytokines, which stimulate cell cycle re-entry and metastasis. This inflammatory reactivation of dormant cells is a critical driver of cancer recurrence [19, 187]. Metabolic stress also plays a crucial role in cancer progression through alterations and bioenergetic adaptations that lead to therapy resistance. Notably, stress hormones can elicit immune responses that reactivate dormant cancer cells, resulting in tumor recurrence [22, 188]. These hormones, including cortisol, can stimulate pathways that promote tumor cell survival and re-entry into the cell cycle, thereby facilitating the reawakening of dormant cells and fueling disease progression. Thus, the intricate balance between metabolic stress, immune signaling, and tumor dormancy plays a crucial role in dictating the fate of the tumor and its propensity for relapse [189]. These factors highlight the complex interplay between genetic, epigenetic, and environmental influences on cancer relapse.

### Role of the TME

The TME significantly contributes to tumor relapse by offering a nurturing environment that enables dormant cancer cells to survive and ultimately resumes their growth. Various components of the TME, such as stromal cells, immune cells, ECM, and soluble factors, interact with cancer cells to modulate their behavior and fate.

Stromal cells within the TME are non-cancerous and provide structural support [190, 191]. These cells, such as CAFs and MSCs, secrete diverse growth factors, cytokines, and ECM components that promote tumor cell survival and proliferation [190]. For instance, studies have demonstrated that bone marrow-derived MSCs can be transformed into CAF-like cells under the influence of factors such as TGF-β secreted by tumor cells. This transformation enhances the pro-tumorigenic properties of these stromal cells, enabling them to support tumor growth and metastasis through ECM remodeling and provision of essential growth factors [143, 192]. These stromal cells can also induce changes in the ECM architecture to create a niche that facilitates cancer cell dormancy while protecting them from immune surveillance and therapeutic interventions [193].

Furthermore, immune cells within the TME, including tumor-infiltrating lymphocytes, MDSCs, and Tregs, exhibit dual roles in tumor relapse. While certain immune cells, such as cytotoxic T cells and NK cells, are capable of recognizing and eliminating dormant cancer cells. Tregs and MDSCs can inhibit anti-tumor immune responses while promoting tumor growth and relapse. However, cancer cells can evade the immune system, establishing a delicate equilibrium wherein various types of immune cells may facilitate tumor growth, spread, and treatment resistance. For instance, the immune attack by CD8^+^ T cells is tempered by a suppressive group of CD4^+^ T cells known as Tregs, characterized by the expression of cluster of differentiation 25 and forkhead box protein P3, which regulate immune tolerance [194]. This suppression mediated by Tregs plays a critical role in tumor dormancy, as Tregs help maintain immune quiescence, enabling cancer cells to evade immune surveillance during periods of dormancy. By inhibiting the activation and proliferation of cytotoxic T cells and NK cells, Tregs result in the establishment of an immunosuppressive microenvironment, allowing dormant tumor cells to persist in a non-proliferative state for extended periods. The balance between immune activation and suppression is essential for tumor dormancy, as Tregs and their associated mechanisms prevent the immune system from eradicating cancer cells that may otherwise remain dormant but eventually reawaken and give rise to recurrence [45, 195]. Thus, the role of Tregs in regulating immune tolerance is not only fundamental to preventing tumor rejection but also to facilitating the long-term survival of dormant tumor cells within the TME.

ECM elements, encompassing proteins and carbohydrates, furnish structural support and biochemical signals to cells, thereby shaping tissue integrity and influencing cell behavior. Within the TME, ECM components such as collagen, fibronectin, and hyaluronic acid offer both structural support and signaling cues that regulate cancer cell behavior. Abnormal ECM remodeling, characterized by increased stiffness and cross-linking, can promote cancer cell survival and proliferation while contributing to therapy resistance and tumor relapse [196, 197]. The interaction between ECM components and cancer cells can activate signaling pathways that confer resistance to treatments [197, 198].

Moreover, soluble factors released by cells within the TME, including growth factors, chemokines, and cytokines, can modulate various signaling pathways involved in cancer cell dormancy and relapse. For example, cells within the TME contribute to therapy resistance by releasing factors such as IL-6, hepatocyte growth factor, FGF, and TGF-β, as well as ECM proteins like integrins. These elements trigger multiple pathways that facilitate tumor survival during treatment [199].

### Metabolic adaptations

Metabolic adaptations represent crucial mechanisms underlying tumor relapse, enabling cancer cells to survive and proliferate under adverse conditions. During dormancy and relapse, cancer cells frequently undergo metabolic reprogramming to meet their energy demands and biosynthetic requirements [200].

Cancer cells may adopt a quiescent metabolic state characterized by reduced glycolysis and oxidative phosphorylation during dormancy. This metabolic dormancy enables cells to survive in nutrient-deprived environments by minimizing energy expenditure and maintaining cellular homeostasis [121]. However, upon reactivation, cancer cells undergo metabolic reprogramming to facilitate rapid proliferation and survival. This reprogramming involves increased glucose uptake, aerobic glycolysis (Warburg effect), and enhanced glutamine metabolism to meet the heightened energy and biosynthetic demands of proliferating cells [200, 201]. Additionally, cancer cells may utilize alternative nutrient sources, such as fatty acids and amino acids, to fuel their metabolic pathways and support cell growth. These metabolic adaptations empower cancer cells to overcome nutrient limitations and promote tumor relapse [202, 203].

Furthermore, the metabolic interactions between cancer cells and the TME play a pivotal role in tumor relapse. The metabolic crosstalk between cancer cells and stromal components, such as CAFs and immune cells, can influence nutrient availability and metabolic signaling pathways, thereby shaping the metabolic landscape of the TME and promoting tumor relapse [204]. Moreover, the metabolic alterations induced by therapeutic interventions, such as chemotherapy and targeted therapy, can contribute to tumor relapse by selecting cancer cells that exhibit enhanced metabolic plasticity and drug resistance. Resistance to therapy frequently involves metabolic adaptations that enable cancer cells to survive and proliferate in the presence of cytotoxic agents [205, 206].

### CSCs and relapse

CSCs have emerged as crucial contributors to tumor relapse due to their distinctive properties of self-renewal, differentiation, and therapy resistance. CSCs represent a subpopulation of cancer cells with stem cell-like characteristics, being capable of initiating and sustaining tumor growth [207, 208]. During periods of tumor dormancy, CSCs may enter a quiescent state, remaining dormant for extended durations while preserving their tumorigenic potential. This dormancy enables CSCs to evade conventional cancer therapies that primarily target rapidly proliferating cells and allows them to survive in adverse microenvironments [12, 13]. Upon reactivation, CSCs can drive tumor relapse by initiating the formation of new tumors or repopulating existing ones [207]. Additionally, CSCs possess intrinsic mechanisms of therapy resistance, such as enhanced DNA repair capacity, drug efflux pumps, and anti-apoptotic pathways, which enable them to withstand chemotherapy and radiation therapy [209, 210]. Moreover, CSCs exhibit phenotypic plasticity, allowing them to transition between quiescent and proliferative states in response to environmental cues and therapeutic interventions. This plasticity enables CSCs to adapt to fluctuating microenvironmental conditions and promote tumor relapse by fueling tumor growth and metastasis [211, 212].

The interaction between CSCs and TME is also essential for tumor relapse. The TME provides a supportive niche for CSCs by supplying growth factors, cytokines, and ECM components that promote CSC self-renewal and maintenance. Additionally, hypoxic and inflammatory conditions within the TME can further enrich the CSC population and enhance their tumorigenic potential [213, 214]. Furthermore, CSCs may undergo epithelial-to-mesenchymal transition (EMT), a process associated with increased invasiveness, metastasis, and therapy resistance. EMT facilitates the dissemination of CSCs from primary tumors, allowing them to survive in circulation and establish metastatic colonies at distant sites, thereby contributing to tumor relapse and disease progression [215].

## Signaling pathways in tumor dormancy and relapse

**Fig. 2:**
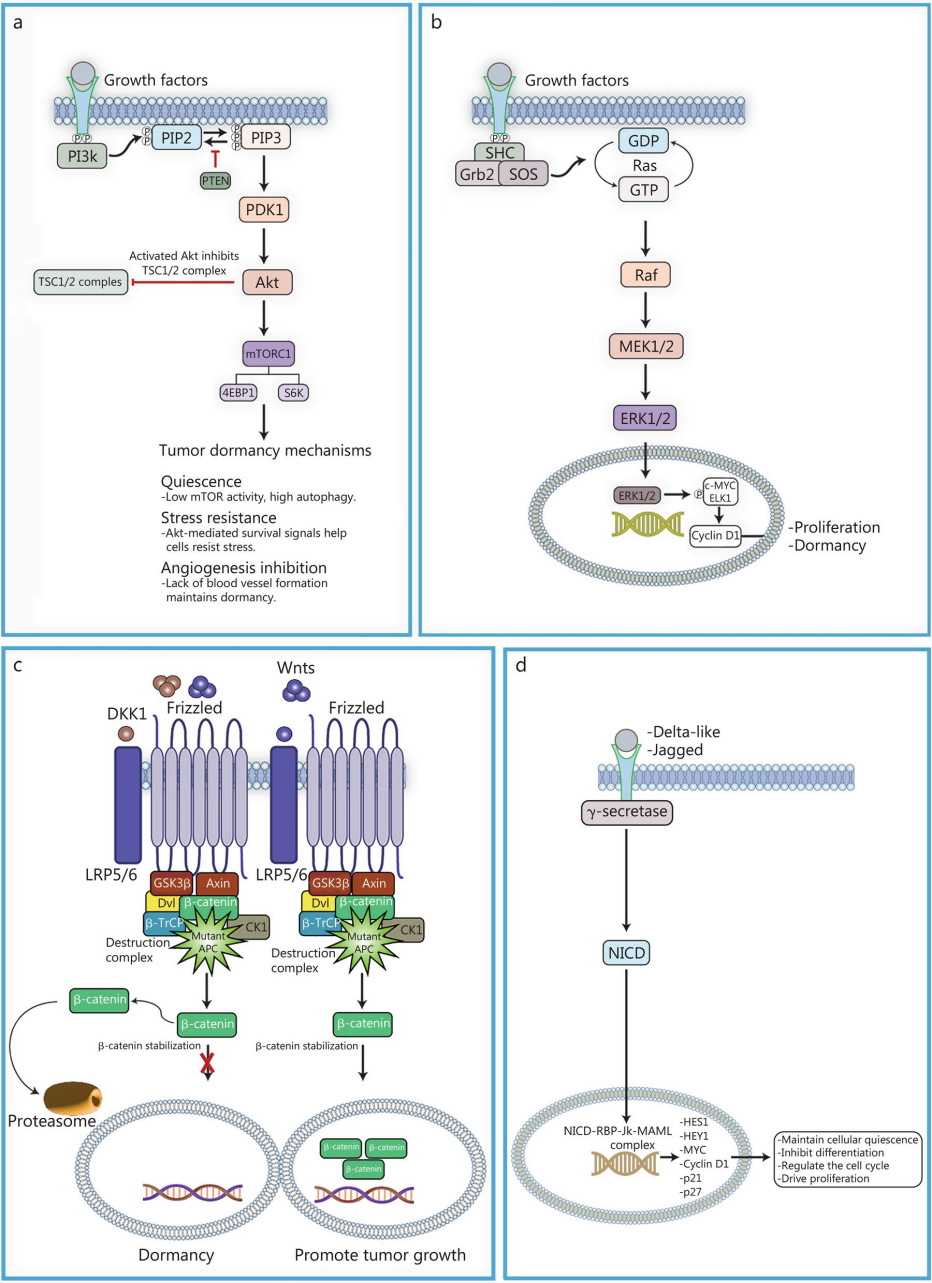
Roles of phosphoinositide 3-kinase/protein kinase B/mechanistic target of rapamycin (PI3K/Akt/mTOR), mitogen-activated protein kinase/extracellular signal-regulated kinase (MAPK/ERK), and Wnt/β-catenin signaling pathways in tumor dormancy and reactivation. **a** Growth factors such as epidermal growth factor and insulin-like growth factor bind to their receptors, causing receptor dimerization and autophosphorylation, which activates PI3K. PI3K converts phosphatidylinositol 4,5-bisphosphate (PIP2) to phosphatidylinositol 3,4,5-trisphosphate (PIP3), recruiting Akt to the cell membrane, where it is phosphorylated by 3-phosphoinositide-dependent protein kinase-1 (PDK1) and mechanistic target of rapamycin complex 2 (mTORC2), leading to its activation. Activated Akt inhibits the tuberous sclerosis complex 1 and 2 (TSC1/2) complex, activating mTORC1, which promotes protein synthesis and cell growth via downstream targets like S6 kinase (S6K) and eukaryotic translation initiation factor 4E-binding protein 1(4EBP1). Phosphatase and tensin homolog (PTEN) negatively regulate this pathway by dephosphorylating PIP3 back to PIP2, acting as a tumor suppressor. The dysregulation of PTEN often results in unchecked PI3K/Akt signaling, contributing to tumorigenesis. Under hypoxic conditions or other stressors, pathway modulation occurs, leading to decreased mTOR activity and enhanced autophagy. These adaptations facilitate tumor dormancy, characterized by quiescent cells exhibiting stress resistance and reduced metabolic demands. In tumor dormancy, pathway modulation leads to low mTOR activity and high autophagy, facilitating cell quiescence, stress resistance, and inhibition of angiogenesis. Additionally, the inhibition of angiogenesis through suppressed VEGF signaling maintains a non-proliferative state by limiting nutrient and oxygen supply. Akt-mediated survival signals allow tumor cells to evade apoptotic cues, and low mTOR activity shifts the balance toward catabolic processes, helping cells resist stress while remaining dormant. **b** The MAPK/ERK pathway is activated by growth factors, e.g., epidermal growth factor (EGF), transforming growth factor-α through Receptor Tyrosine Kinases (RTKs) like epidermal growth factor receptor (EGFR), initiating a cascade that involves adaptor proteins growth factor receptor-bound protein 2 (Grb2) and son of sevenless (SOS), Ras activation, Raf stimulation, and phosphorylation of mitogen-activated protein kinase kinase 1 and 2 (MEK1/2) and ERK1/2. ERK1/2 translocates to the nucleus to phosphorylate transcription factors like c-MYC and ETS-like protein 1 (ELK1), promoting cell cycle progression via cyclin D1 upregulation and integrating survival signals and environmental cues. **c** The Wnt/β-catenin pathway involves Wnt ligands binding to frizzled and low-density lipoprotein receptor-related protein 5 or 6 (LRP5/6) receptors, activating dishevelled (Dvl) and inhibiting the β-catenin destruction complex [adenomatous polyposis coli (APC), Axin, glycogen synthase kinase 3 beta (GSK-3β), casein kinase 1 (CK1)], leading to β-catenin stabilization and nuclear translocation, where it interacts with T cell factor/lymphoid enhancer factor transcription factors to activate gene expression. In dormant tumor cells, high levels of the Wnt inhibitor Dickkopf1 (DKK1) secreted by the microenvironment result in active β-catenin degradation and maintenance of the dormant state. **d** Ligand binding to the NOTCH receptor triggers proteolytic cleavage, releasing the NOTCH intracellular domain (NICD), which translocates to the nucleus. NICD binds to the transcription factor RBP-Jκ, displacing corepressors and forming a transcriptional activation complex with coactivators like MAML and p300/CBP. This complex promotes the expression of target genes such as HES1, HEY1, Myc, cyclin D1, p21, and p27, which regulate cellular quiescence, stress resistance, and cell cycle arrest, contributing to tumor dormancy

The intricate signaling pathways that regulate tumor dormancy and relapse, as shown in Fig. 2, involve the PI3K/Akt/mTOR, the mitogen-activated protein kinase (MAPK)/ERK, Wnt/β-catenin, and Notch signaling pathways. These pathways are pivotal in maintaining the delicate balance between cellular quiescence and proliferation. Below, we have elaborated on several key pathways related to tumor dormancy and relapse.

### PI3K/Akt/mTOR pathway

The PI3K/Akt/mTOR pathway regulates numerous cellular processes, including growth, survival, and metabolism. Dysregulation of this pathway may result in cancer progression and recurrence [216, 217]. The cascade initiates with the activation of PI3K, which can be triggered by various signals such as growth factors, cytokines, and hormones [218]. Upon activation, PI3K catalyzes the conversion of phosphatidylinositol 4,5-bisphosphate (PIP2) into phosphatidylinositol 3,4,5-trisphosphate (PIP3). PIP3 functions as a docking site for several proteins, particularly Akt. Subsequently, Akt is recruited to the plasma membrane where it undergoes phosphorylation and activation by 3-phosphoinositide-dependent protein kinase 1 and mTORC2, leading to the phosphorylation of multiple downstream targets that govern cell survival, proliferation, and metabolism [218, 219].

Activation of Akt promotes cell survival by inhibiting pro-apoptotic factors, thereby facilitating an anti-apoptotic signaling cascade that enables dormant cells to resist programmed cell death. In the context of chronic hypoxia, suppression of Akt activity is necessary to induce dormancy and ensure the survival of cancer cells [220]. Furthermore, Akt modulates the cell cycle by phosphorylating key regulators such as p21 and p27, effectively preventing cell cycle progression and aiding in the maintenance of cellular dormancy [221, 222]. Additionally, Akt plays a critical role in regulating glucose metabolism by enhancing glucose uptake and promoting glycolysis to meet the energy demands of dormant cells [223, 224]. The activation of Akt can significantly increase glucose absorption in cancer cells through modulation of glucose transporter 1, a principal glucose transporter. Sustained activity of Akt leads to elevated levels of glucose transporter 1 on the cell surface, thus facilitating enhanced glucose entry. This oncogenic activation within the PI3K/Akt pathway drives increased glucose uptake [225]. Autophagy is a vital cellular process responsible for degrading and recycling damaged organelles, misfolded proteins, and other cellular debris to maintain homeostasis and respond effectively to stressors. This pathway also influences autophagy as another mechanism for survival. mTOR serves as a downstream target of Akt that inhibits autophagy when active. However, reduced mTOR activity during dormancy can trigger autophagy processes that assist cells in surviving under low-nutrient conditions [226, 227].

The PI3K/Akt/mTOR signaling pathway also plays a significant role in tumor relapse. Alterations in the microenvironment, such as enhanced nutrient availability and improved oxygen levels, can alleviate stress conditions that maintain cellular dormancy and reactivate this pathway [228]. Genetic and epigenetic modifications may further augment pathway activity. For instance, mutations in *PTEN*, a negative regulator of PI3K, result in sustained Akt activation [169, 229]. PTEN acts as a tumor suppressor by dephosphorylating PIP3 back to PIP2, thereby inhibiting the signaling cascade. In the context of tumor dormancy, PTEN plays a critical role in maintaining a balance between cell survival and quiescence, thus preventing uncontrolled cell division and tumor progression. Loss or dysfunction of PTEN disrupts this equilibrium, leading to aberrant activation of Akt. This activation promotes cell survival, induces angiogenesis, and can reawaken dormant tumor cells, facilitating their transition into an actively growing and metastatic state. Consequently, PTEN serves as a essential tumor suppressor, and its loss may be a key event in the escape from dormancy and subsequent tumor relapse [230]. The PI3K/Akt/mTOR pathway is frequently associated with resistance to cancer therapies [218, 231], enabling dormant cells to survive initial treatments and later contributing to disease relapse [232]. Hyperactivation of this pathway has been linked with drug resistance and cancer progression [233]. This survival mechanism not only enables dormant cells to persist during initial treatments but also facilitates their reactivation upon therapy withdrawal or mutation, leading to tumor re-emergence. Furthermore, the pathway supports tumor dormancy by influencing the TME through ECM remodeling and stress responses, thereby maintaining the delicate balance between dormancy and reactivation. This complexity complicates the development of effective treatment strategies [234, 235]. Currently, several pharmacological agents targeting the PI3K/Akt/mTOR axis are under investigation in clinical trials aimed at combining these agents with standard therapies to overcome acquired resistance across various cancer types [231, 236].

Targeting the PI3K/Akt/mTOR signaling pathway holds substantial therapeutic potential by disrupting survival mechanisms in dormant cells. PI3K inhibitors, such as idelalisib and alpelisib, reduce survival signals, while Akt inhibitors like ipatasertib disrupt multiple downstream pathways [237, 238]. Additionally, mTOR inhibitors such as rapamycin promote autophagy and decrease cell proliferation [239, 240]. These inhibitors not only help maintain dormancy but also sensitize cells to other treatments, including chemotherapy. The combination of these pathway inhibitors with conventional therapies can enhance their efficacy, aiming to eliminate both proliferating and dormant tumor cells.

### MAPK/ERK pathway

The MAPK/ERK pathway starts with the activation of receptor tyrosine kinases (RTKs) by external signals such as growth factors. Once activated, RTKs recruit and activate Ras, a small GTPase. Ras subsequently activates the kinase Raf, which in turn activates MEK. MEK phosphorylates and activates ERK, the final kinase in the cascade. ERK translocates to the nucleus and phosphorylates various transcription factors. These transcription factors regulate genes related to cell proliferation, survival, and differentiation.

The MAPK/ERK pathway plays a crucial role in tumor dormancy, it can largely maintain and disrupt the dormant state by regulating the cell cycle [241]. Low ERK activity can keep cells quiescent or dormant by reducing the expression of cyclins and other proteins that regulate the cell cycle [241]. Their downregulation leads to cell cycle arrest, a key feature of dormancy [242]. ERK activity is modulated by diverse factors, including cellular stress and microenvironmental signals [243]. For instance, hypoxic conditions, which are common in dormant tumor niches, can reduce ERK signaling, thereby facilitating the maintenance of the dormant state [244]. Conversely, intermittent or low-level ERK activation can support cell survival without triggering proliferation. This balance between dormancy and survival is critical for the fate of dormant tumor cells [245]. The MAPK/ERK pathway also affects metabolic adaptation in dormant cells. Low ERK activity is associated with decreased anabolic metabolism, which enables cells to survive in nutrient-poor conditions by reducing their energy demands [246]. For example, the ERK(MAPK)/p38(SAPK) activity ratio can predict whether cells will proliferate or enter a state of dormancy, suggesting that the ERK pathway plays a significant role in determining the metabolic state of cells [241].

Moreover, ERK signaling can regulate autophagy. Autophagy supports cell survival during dormancy, providing another link between ERK signaling and the maintenance of the dormant state [247]. For instance, growth factor exposure can increase the interaction between ERK components and autophagy-related proteins, facilitating the phosphorylation of ERK and promoting autophagy [248, 249]. Recent research highlights how the surrounding environment influences the destiny of DTCs. For instance, in a conducive setting like the lungs, DTCs can assimilate and respond to signals that encourage growth, like those from fibronectin. This results in the activation of mitogenic signals, characterized by a high ERK to low p38 ratio, which stimulates the proliferation of DTCs [250].

The reactivation of the MAPK/ERK pathway is also important in tumor relapse. Increased ERK activity can drive cells out of a dormant state and back into active proliferation [241]. Various factors, including growth factors or changes in the microenvironment, can trigger this by activating RTKs, leading to enhanced ERK signaling. Mutations in pathway components can also lead to sustained ERK activation. For instance, mutations in *Ras* or *BRAF* (a *Raf* isoform) are common in many cancers. These mutations result in continuous ERK signaling, bypassing normal regulatory mechanisms [251, 252], leading to the reawakening of dormant cells [252]. Additionally, stress responses can influence ERK activation. For example, tissue damage or inflammation release growth factors and cytokines [253, 254]. These factors can activate the MAPK/ERK pathway, potentially triggering dormant cells to resume growth [241, 255].

Moreover, several recent research have discussed its role in tumor relapse. For instance, a recent study suggested that hyperactivation of the MAPK signaling is associated with over 40% of human cancer cases [256]. This signaling promotes cellular overgrowth and enables cells to overcome metabolic stress. Another study discussed the implications of MAPK signaling in cancer therapy response [257]. Similarly, whole-genome sequencing of paired diagnostic and relapse neuroblastomas revealed clonal evolution and a significant accumulation of somatic mutations in relapse tumors, with a median of 29 mutations unique to the relapse samples. Notably, 78% of relapse tumors exhibited mutations predicted to activate the RAS/MAPK signaling pathway, some of which were present only in relapse samples, indicating clonal enrichment. In neuroblastoma cell lines, these activating mutations were found at a high frequency (61%) and were associated with sensitivity to MEK inhibition both in vitro and in vivo [258]. Moreover, activation of p38 MAPK plays a critical role in tumor relapse in HNSCC by maintaining the CSC phenotype, leading to therapy resistance and impaired DNA damage repair. Inhibition of p38 MAPK resulted in decreased expression of CSC marker, increased chemosensitivity, reduced migration, and impaired sphere-forming ability, along with enhanced DNA damage response, as indicated by elevated comet olive tail moment and gamma-H2A histone family member X (γ-H2AX) accumulation. These findings suggest that p38 MAPK activation is involved in tumor recurrence, highlighting its potential as a therapeutic target for overcoming relapse in HNSCC [259]. Furthermore, it was found that upon experimental suppression of MAPK, compensatory mechanisms are activated. Furthermore, another study mentioned that the main mechanism of resistance to Raf or MEK inhibitors is the reactivation of ERK signaling [260].

Therefore, therapies targeting the MAPK/ERK pathway, such as *BRAF* and MEK inhibitors (e.g., vemurafenib, dabrafenib, trametinib, and selumetinib), can help control tumor growth and maintain dormancy. Specifically, *BRAF* inhibitors target mutant *BRAF* to regulate ERK activation, while MEK inhibitors block downstream ERK activation, thereby preventing cell cycle progression [261, 262]. These inhibitors, when combined with treatments such as immunotherapy, can improve treatment efficacy. Biomarkers like phosphorylated ERK levels can monitor ERK activity, providing valuable information on tumor status, treatment response, and potential relapse.

### Wnt/β-catenin pathway

The Wnt/β-catenin pathway, which is vital for regulating cell proliferation, differentiation, and survival, has a significant impact tumor dormancy and relapse. The pathway initiates with the binding of Wnt proteins to Frizzled and low-density lipoprotein receptor-related protein 5 or 6 co-receptors, activating dishevelled proteins. This activation inhibits the destruction complex glycogen synthase kinase 3 beta, Axin, and adenomatous polyposis coli (APC), which normally degrades β-catenin, allowing β-catenin to accumulate and translocate to the nucleus. In the nucleus, β-catenin interacts with T cell factor/lymphoid enhancer-binding factor (TCF/LEF) transcription factors to activate genes that promote cell proliferation, survival, and differentiation [263, 264].

Low or intermittent Wnt signaling can maintain cells in a dormant state by regulating genes that control cell cycle arrest and apoptosis [265]. This pathway affects cell cycle regulators such as cyclin D1 and c-MYC, and reduced Wnt activity leads to lower levels of these proteins, contributing to cell cycle arrest and quiescence [266, 267]. Dormant tumor cells often exhibit stem-like properties, and Wnt/β-catenin signaling is crucial for maintaining these characteristics by regulating stemness genes, enabling dormant cells to retain the ability to re-enter the cell cycle upon activation. Additionally, Wnt signaling helps dormant cells adapt to low-nutrient conditions by modulating metabolic pathways, such as glycolysis and oxidative phosphorylation, thus supporting efficient energy production and cell survival [268, 269].

The reactivation of the Wnt/β-catenin pathway is a critical factor in tumor relapse, as enhanced Wnt signaling can prompt dormant cells to re-enter the cell cycle and proliferate [270]. Various factors, including changes in the TME such as increased availability of growth factors and nutrients, can trigger this reactivation by activating Wnt signaling and disrupting dormancy [271, 272]. Genetic alterations, such as mutations in APC or Axin that prevent the formation of the destruction complex, result in continuous β-catenin accumulation [273, 274]. Epigenetic changes can also upregulate Wnt pathway genes, contributing to its reactivation. Additionally, crosstalk with other pathways like PI3K/Akt and MAPK/ERK can strengthen Wnt signaling, amplifying survival and proliferative signals, and awakening dormant cells [270, 275].

Targeting the Wnt/β-catenin pathway offers potential therapeutic strategies for managing tumor dormancy and preventing relapse. Several drugs are designed to inhibit Wnt signaling. Small molecules like ICG-001 disrupt the interaction between β-catenin and TCF/LEF, thereby preventing transcription of target genes [276, 277]. The combination of Wnt inhibitors with other treatments can improve efficacy. For instance, combining Wnt inhibitors with chemotherapy or immunotherapy can assist in eliminating dormant and actively proliferating cells [278, 279]. Additionally, monitoring the activity of the Wnt/β-catenin pathway can help assess tumor status [263, 280]. Biomarkers such as β-catenin levels and the expression of Wnt target gene can indicate pathway activation, guiding treatment decisions and predicting relapse [263, 265].

### Notch signaling pathway

The Notch signaling pathway is a highly conserved mechanism essential for cellular processes such as proliferation, differentiation, and apoptosis. The pathway involves transmembrane receptors (Notch receptors) and ligands (delta-like and Jagged families), where activation occurs through direct cell-to-cell contact. This interaction triggers proteolytic cleavage of the Notch receptor, releasing the Notch intracellular domain, which then translocates to the nucleus to regulate target gene expression.

The Notch pathway plays a significant role in tumor dormancy, particularly in maintaining quiescent states of cancer cells. Low or intermittent Notch signaling can induce cell cycle arrest and promote cellular quiescence by regulating the expression of genes that control these processes [281, 282]. For instance, a study on malignant gliomas, particularly glioblastoma, revealed that Notch signaling correlates with an undifferentiated tumor cell state and facilitates the escape from tumor dormancy, leading to recurrence and progression after treatment [283]. Similarly, Notch signaling plays a pivotal role in tumor dormancy and recurrence in breast cancer. Study has demonstrated that Notch signaling is upregulated in residual tumor cells following therapy, particularly after inhibition of the HER2/neu pathway, and remains activated in a subset of dormant tumor cells. This activation contributes to tumor recurrence upon reactivation [284]. Additionally, Notch2 signaling has been implicated in cellular dormancy, particularly within the bone microenvironment, where it influences the survival and mobilization of dormant breast cancer cells. Notch2^high^ cells exhibit a stem-like phenotype, tumor-initiating ability, and a survival advantage, highlighting Notch signaling as a key regulator of dormancy [285]. This suggests that interventions to reduce or block Notch expression could trigger tumor cell differentiation, thereby facilitating treatment [283]. Additionally, Notch signaling assists in regulating the balance between stem cell-like properties and differentiation, allowing dormant cells to retain the potential to re-enter the cell cycle upon activation [286, 287]. This pathway also modulates cellular metabolism, which is crucial for the survival of dormant cells in nutrient-deprived environments. By influencing glycolysis and oxidative phosphorylation, Notch signaling helps cells manage energy production efficiently, supporting their survival during dormancy [288].

Conversely, the reactivation of Notch signaling contributes to tumor relapse by promoting the exit from dormancy and initiating cell proliferation. Factors such as changes in the TME, including increased levels of growth factors and cytokines, can activate Notch signaling and drive dormant cells back into the cell cycle [289, 290]. Moreover, genetic and epigenetic alterations in Notch pathway components can lead to aberrant pathway activation, further fueling tumor relapse. For instance, mutations in Notch-related genes or dysregulated upstream pathways result in continuous Notch signaling, bypassing normal regulatory mechanisms [291]. This persistent signaling can lead to the reawakening of dormant cells.

Therapeutically targeting the Notch signaling pathway poses challenges and opportunities in cancer treatment. While Notch inhibitors have shown potential in preclinical studies for preventing tumor growth and metastasis, off-target effects and dose-limiting toxicities have limited their application in clinical settings [292, 293]. However, combination therapies that target multiple signaling pathways, including Notch, may hold the potential for effectively managing tumor dormancy and preventing relapse.

## Detection and monitoring of dormant tumors

**Fig. 3:**
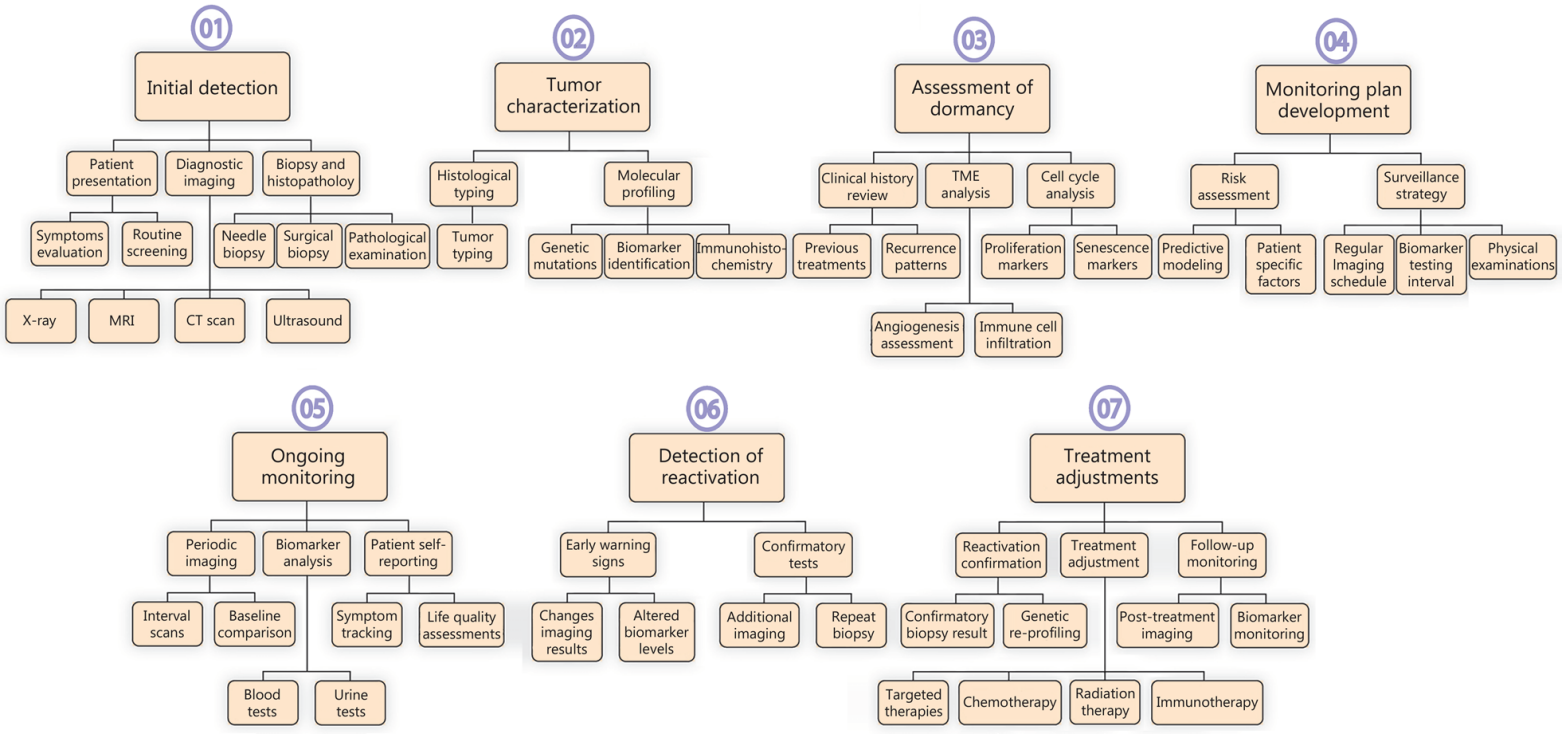
Detection and monitoring process for dormant tumors. The flow chart illustrates a comprehensive process for the detection and monitoring of dormant tumors, encompassing various stages from initial detection to potential reactivation. It begins with initial detection modalities such as diagnostic imaging and biopsy, followed by tumor characterization through histological typing and molecular profiling. The assessment of dormancy involves reviewing clinical history, analyzing the tumor microenvironment (TME), and evaluating cell cycle status. Subsequently, a monitoring plan is developed based on risk assessment and surveillance strategies, which include periodic imaging, biomarker analysis, and patient self-reporting. Reactivation detection involves identifying early warning signs and conducting confirmatory tests, leading to response and treatment adjustments such as targeted therapies or chemotherapy. Continuous follow-up monitoring ensures ongoing assessment of treatment efficacy and tumor status. MRI magnetic resonance imaging, CT computed tomography

Detecting and monitoring dormant tumors poses a significant challenge in oncology. Dormant tumors are characterized by their absence of growth and minimal metabolic activity, making them difficult to identify using conventional imaging and diagnostic techniques. Nevertheless, advancements in technology and research are offering new approaches to detect and monitor these elusive cancer cells. Here, we present a detailed flow chart outlining the steps for detecting and monitoring dormant tumors, which is crucial for oncology (Fig. 3). It guides clinicians in patient management and helps researchers study tumor dormancy.

### Traditional imaging

Traditional imaging methods such as X-ray, computed tomography (CT), and magnetic resonance imaging (MRI) are often ineffective in detecting dormant tumors due to their small size and low metabolic activity. However, advancements in imaging technologies are improving this capability. For example, positron emission tomography (PET) employs a radioactive drug referred to as a tracer to disclose the metabolic or biochemical function of tissues and organs. Tracers accumulate in body regions that exhibit increased metabolic or biochemical activity, frequently identifying illness sites. On PET scan, cancer cells are highlighted as luminous areas because of their elevated metabolic activity compared to normal cells. Nonetheless, careful interpretation of PET scan is necessary, as benign conditions may mimic cancer, and certain cancers may not be visible [294, 295]. Also, certain types of cancer do not consume a significant amount of glucose, resulting in the possibility that a negative PET scan may miss certain cancerous tumors. Moreover, MRI provides detailed images of soft tissues, making it useful for detecting structural changes associated with dormant tumors. Enhanced MRI methods outperform traditional MRI in determining the size of tumors, predicting their grade, and monitoring the effectiveness of treatments. Additionally, approaches such as diffusion-weighted imaging (DWI) and dynamic contrast-enhanced MRI (DCE-MRI) yield valuable information about tumors’ blood supply and cellularity. These techniques have the potential to identify dormant tumors based on changes in the TME [296]. In recent years, the integration of artificial intelligence with these imaging techniques has shown promising outcomes. For instance, a study demonstrated the application of a deep learning model, ResNet50 improved with Grad-CAM, for brain tumor detection in MRI images, achieving a testing accuracy of 98.52% [297]. The fusion of high precision and clarity in this context has significant implications for medical diagnostics, paving the way for the development of tumor detection instruments that are both more reliable and easier to understand [297]. It is important to note that although these advanced imaging techniques have considerably improved our ability to detect and diagnose tumors, they are not infallible and should be utilized in conjunction with other diagnostic tests and clinical information for the most accurate results.

### Biomarkers for dormant tumors

Biomarkers are molecules that can be found in blood, other body fluids, or tissues and can indicate the presence of cancer. The identification of biomarkers specific to dormant tumors is crucial for early detection and monitoring.

CTCs are cancer cells detached from the primary tumor and traveling through the bloodstream. Liquid biopsy techniques can detect and analyze CTCs, offering insights into the tumor’s genetic and phenotypic traits, and helping monitor tumor dynamics, including early signs of dormancy or reactivation [298, 299]. The identification of CTCs typically relies on specific molecular markers, such as the commonly used epithelial cell adhesion molecule, although these markers vary across cancer types. CTCs exhibit characteristics associated with increased metastatic potential, similar to those witnessed in the EMT and stem cell-like properties. However, only a minority of CTCs succeed in surviving and leading to metastasis, highlighting the crucial role of their interactions with the challenging environment in the bloodstream. Advances in single-cell sequencing have shed light on the genetic and expression profiles of CTCs [299]. Recently, immunotherapy has emerged as a key element in cancer therapy. When employed in conjunction with traditional treatments such as surgery, radiation, and chemotherapy, it seems to enhance the survival outcomes of patients [202]. Therefore, studying the connection between CTCs and immunotherapy is essential for improving cancer treatment.

Tumor cells release extracellular vesicles (EVs), including a subset called exosomes, into the bloodstream. These vesicles carry proteins, RNA, and DNA derived from tumor cells and serve as biomarkers for cancer detection [300, 301]. These are uniquely designed to facilitate communication between cells by transmitting genetic material, which encompasses coding and non-coding RNAs, to target cells [300]. Consequently, exosomes and microvesicles are integral to biological functions, governing both healthy and diseased states through gene regulatory frameworks and epigenetic modifications. Analyzing the molecular content of exosomes can provide insights into the tumor’s dormant state and its potential to reactivate [300, 301]. Exosomes play a pivotal role in numerous physiological functions, including immune regulation [302, 303], tissue regeneration [304, 305], sustaining stem cells [306, 307], and central nervous system signaling [308, 309], as well as in disease mechanisms related to heart conditions [310, 311], neurodegenerative disorders [312, 313], cancers [314, 315], and inflammatory responses [316, 317]. Their potential for clinical use, both as indicators for diagnosis and as vehicles for treatment, has attracted significant attention. The biocompatible nature of exosomes and their dual-layered lipid composition, which protects the genetic material they carry from deterioration, renders them promising candidates for use in therapy delivery systems [301]. Their diminutive dimensions and specific membrane structure enable them to cross significant biological barriers, such as the blood–brain barrier.

### Advanced imaging techniques

Fluorescence imaging, particularly with probes that light up specific tumor-related molecules or cells, has emerged as a key tool in cancer detection. It offers sharp, high-contrast images and can track dormant cells in early studies. Dyes such as cyanine and porphyrin, renowned for their intense fluorescence, facilitate the clear distinction of targets from their surroundings [318, 319]. The technology also includes probes that activate under certain conditions, leading to the early identification of cancer [320, 321].

Bioluminescence imaging is primarily utilized in research settings and involves genetically modifying tumor cells to express bioluminescent proteins. This technique allows real-time tumor monitoring of growth in animal models. This can provide valuable insights into dormancy mechanisms. Using bioluminescence imaging, researchers could conduct highly quantitative measurements on the tumor regression and reemergence rate [322, 323]. Based on the signal intensity, numerous tumor cells appeared to be proliferatively expanding upon *MYC* reactivation [324, 325].

### High-throughput technologies

High-throughput sequencing and proteomic analyses can identify genetic and protein changes related to tumor dormancy. Next-generation sequencing (NGS), also referred to as high-throughput sequencing, is a cutting-edge technology employed to determine the sequence of nucleotides in a piece of DNA or RNA. Unlike traditional Sanger sequencing, NGS enables the rapid sequencing of extensive stretches of DNA, making it feasible to sequence entire genomes, exomes, or transcriptomes quickly and efficiently [326, 327]. NGS permits the sequencing of multiple genes at an extremely high depth of coverage. It can furnish comprehensive genetic profiles of tumors [328]. Researchers can identify mutations and pathways associated with dormancy by comparing genetic alterations in primary and dormant tumors [329, 330]. This information assists in the development of targeted therapies and monitoring strategies.

Proteomics represents a significant advancement in scientific research. It encompasses the comprehensive analysis of proteomes, the entire set of proteins expressed or modified by a biological entity [331, 332]. Identifying proteins explicitly expressed in dormant cells can offer biomarkers for detection and targets for therapy [333, 334]. Mass spectrometry and other high-throughput techniques are revolutionizing the analysis of proteins from tumors and bodily fluids. These methods, including advanced tissue microarrays and single-cell analysis, are pivotal in biomedical research and healthcare. They provide insights into complex medical challenges and hold great promise for fundamental research, cancer prognosis, tailored treatments, and new drug development. Moreover, single-cell sequencing of CTCs enables the comparison of genomic, transcriptomic, and epigenetic variations across individual cells from blood, primary tumors, metastases, and lymph nodes, minimizing the impact of tumor diversity [335]. This gives a new viewpoint for identifying the biological process of tumor occurrence and development.

## Clinical implications of tumor dormancy and relapse

### Diagnostic biomarkers for dormancy and relapse

There are diverse clinical implications of tumor dormancy and relapse. Diagnostic biomarkers for dormancy and relapse are essential for the early detection and monitoring of cancer progression. Molecular biomarkers offer valuable insights into the underlying mechanisms driving dormancy and relapse, facilitating personalized treatment strategies for cancer patients. One type of molecular biomarkers for dormancy and relapse is gene expression profiles. Changes in the expression levels of specific genes associated with dormancy-related pathways, such as cell cycle arrest, apoptosis resistance, and stemness, can indicate dormant or reactivated tumor cells [336, 337]. For example, the upregulation of cell cycle inhibitors such as p27 and p21, and the downregulation of proliferation markers like Ki-67 may signify a dormant state in cancer cells [26, 338]. Conversely, the activation of survival pathways, such as PI3K/Akt and MAPK/ERK [339, 340], or the expression of stem cell markers like CD44 and aldehyde dehydrogenase 1, may imply tumor relapse and progression [341, 342]. Another molecular biomarker approach involves the analysis of CTCs and circulating tumor DNA (ctDNA). CTCs are scarce cancer cells that detached from the primary tumor and enter the bloodstream, while ctDNA consists of tumor-derived DNA fragments released into circulation. The detection and characterization of CTCs and ctDNA can provide real-time information about tumor burden, genetic heterogeneity, and treatment response [343, 344]. Changes in the number and genetic profile of CTCs and ctDNA over time serve as dynamic biomarkers for predicting dormancy, relapse, and treatment outcomes [345, 346]. Additionally, epigenetic biomarkers, such as DNA methylation patterns and histone modifications, show great potential for predicting dormancy and relapse in cancer. Alterations in the epigenetic landscape of tumor cells exert an influence on gene expression patterns and cellular behaviors associated with dormancy and relapse [63, 347]. For instance, hypermethylation of tumor suppressor gene promoters and hypomethylation of oncogene promoters may contribute to tumor dormancy and reactivation, respectively. Similarly, histone modifications such as acetylation and methylation can regulate chromatin accessibility and gene expression dynamics in dormant and proliferating tumor cells [348].

Various imaging techniques can also provide diagnostic biomarkers for dormancy and relapse, offering non-invasive and real-time assessments of tumor status. These imaging modalities provide valuable information about tumor size, location, metabolic activity, and microenvironmental characteristics, facilitating the detection and monitoring of cancer dormancy and relapse. One common imaging technique used in cancer diagnosis is MRI, which can offer detailed anatomical images of tumors, enabling clinicians to assess tumor size, invasion into surrounding tissues, and the presence of metastases. Functional MRI techniques, such as DWI and DCE-MRI, can provide data about tumor cellularity, perfusion, and vascularity, which are relevant to tumor dormancy and relapse [349]. Another extensively employed imaging modality is PET in combination with CT or MRI [350]. PET imaging makes use of radiotracers that are absorbed by metabolically active cells, allowing the visualization of tumor metabolism. This metabolic information help identify regions of tumor dormancy or detect early indications of tumor relapse by evaluating changes in glucose uptake, amino acid metabolism, or hypoxia within the TME. Furthermore, molecular imaging techniques, such as single-photon emission computed tomography and PET with specific radiotracers targeting molecular pathways related to dormancy and relapse, offer insights into tumor biology at the molecular level. For example, PET imaging with radiotracers targeting cell proliferation markers (e.g., [^18^F]fluorothymidine) or hypoxia (e.g., [^18^F]fluoromisonidazole) provides information about tumor dormancy and potential reactivation. Moreover, advanced imaging techniques like diffusion-weighted MRI and diffusion tensor imaging assess tissue microstructure and integrity, providing data about cellular density, tissue organization, and structural connectivity within the tumor and surrounding tissues [351, 352]. These imaging biomarkers help differentiate between dormant and actively proliferating tumor cells and monitor changes in tumor behavior over time.

### Predicting recurrence risk

Predicting the recurrence risk in cancer patients is of crucial significance for guiding treatment decisions and improving outcomes. Genetic profiling offers valuable insights into the molecular characteristics of tumors, facilitating the identification of genetic alterations associated with recurrence risk. One approach for predicting recurrence risk is to analyze the genetic profile of tumors using techniques such as NGS or gene expression profiling. These technologies allow researchers to identify specific genetic mutations, copy number alterations and gene expression patterns that may influence tumor behavior and recurrence. For example, mutations in tumor suppressor genes such as *TP53*, *PTEN*, and *RB1* [353], or oncogenes like *KRAS* and *BRAF* [354], were associated with an increased recurrence risk in various cancer types. Additionally, alterations in DNA repair genes, such as *BRCA1* and *BRCA2*, may impact the response to treatment and predispose patients to recurrence [355].

Gene expression profiling can also provide insights into recurrence risk by assessing the activity of specific molecular pathways involved in tumor progression and metastasis [356]. High expression levels of genes associated with EMT, angiogenesis, and immune evasion have been linked to poor prognosis and an increased recurrence risk in patients with cancer [357, 358]. For example, EMT-related markers such as Vimentin [359, 360], Snail [361, 362], and Twist [363, 364], promote tumor invasiveness and metastasis by facilitating the transition to a mesenchymal phenotype. Similarly, angiogenic factors like VEGF and FGF enhance tumor growth by supporting blood vessel formation [365, 366], while immune evasion mechanisms involving PD-L1 and CTLA-4 allow cancer cells to escape immune surveillance [367, 368]. Together, these molecular changes contribute to tumor progression and resistance to treatment, underscoring their role in adverse clinical outcomes. Furthermore, integrating genetic profiling with clinical and pathological factors enhance the accuracy of recurrence risk prediction models. By combining genetic information with traditional prognostic factors such as tumor stage, grade, and lymph node involvement, clinicians more effectively stratify patients based on their risk of recurrence and tailor treatment strategies accordingly [369, 370]. In some cases, genetic profiling may even uncover actionable targets or predictive biomarkers for targeted therapies or immunotherapy, which assist in preventing recurrence or improving treatment response in high-risk patients [371]. Overall, genetic profiling holds great potential for predicting recurrence risk in patients with cancer by identifying genetic alterations and molecular pathways associated with tumor aggressiveness and metastatic potential.

Liquid biopsies, which entail the analysis of biomarkers present in blood or other bodily fluids, offer a non-invasive and convenient approach to monitoring tumor dynamics and predicting recurrence [372]. One key advantage of liquid biopsies is their ability to capture tumor-derived materials, such as CTCs [373, 374], cell-free DNA (cfDNA) [375], and EVs [376], released into the bloodstream or other bodily fluids. These tumor-derived components in liquid biopsies, such as CTCs, reflect both primary and metastatic tumors. Analyzing CTCs can predict recurrence risk by assessing their presence, number, and molecular traits. High CTC levels are related to a greater risk of recurrence and a poor prognosis, indicating residual disease or metastasis [377, 378]. Similarly, detecting and characterizing cfDNA in liquid biopsies can provide information about tumor burden, genetic mutations, and clonal evolution over time. Changes in cfDNA levels or specific genetic alterations may indicate minimal residual disease, disease progression, or the emergence of treatment-resistant clones, thereby predicting recurrence risk and guiding therapeutic interventions. Furthermore, liquid biopsies present the opportunity for sequential monitoring of recurrence risk over time, enabling clinicians to track disease dynamics, assess treatment response, and detect early signs of recurrence. By integrating liquid biopsy data with clinical and imaging findings, clinicians can formulate personalized surveillance strategies and tailor treatment plans to minimize the risk of recurrence and improve patient outcomes.

### Therapeutic strategies

Therapeutic strategies targeting dormant cancer cells offer promising avenues for preventing tumor relapse. One effective approach is to awaken dormant cancer cells from their quiescent state, rendering them vulnerable to conventional treatments such as chemotherapy and radiation. This strategy takes advantage of the notion that reactivating these cells make them susceptible to therapies that would otherwise be ineffective against them [379]. Another critical strategy focuses on targeting the survival pathways and molecular mechanisms that enable cancer cells to maintain dormancy. By inhibiting key pathways, such as the PI3K/Akt and MAPK/ERK signaling pathways, researchers aim to prevent the reactivation of dormant cells. This could significantly reduce the risk of relapse by disrupting the cellular processes that allow these cells to survive undetected [380, 381]. Additionally, targeting the TME, which provides a supportive niche for dormant cells, can be a promising therapeutic approach [8, 382]. Disrupting the interactions between cancer cells and stromal cells, immune cells, and ECM components may prevent the survival and reactivation of dormant cells. Immunotherapy represents another avenue for targeting dormant cells and preventing relapse. By activating the immune system to recognize and eliminate dormant cancer cells, immunotherapeutic approaches such as checkpoint inhibitors and adoptive cell therapies may potentially prevent relapse and improve long-term outcomes for patients with cancer.

Preventing relapse is a crucial aspect of cancer management and requires targeted therapeutic strategies. One approach involves targeting residual disease and micrometastases to eliminate dormant cancer cells before they become clinically detectable and lead to relapse. Adjuvant therapies, administered subsequent to primary treatment such as surgery or chemotherapy, aim to eradicate residual disease and prevent recurrence. These therapies may include targeted agents, immunotherapy, or maintenance chemotherapy tailored to the tumor’s specific molecular and genetic characteristics. Targeted therapies directed against specific molecular pathways implicated in tumor progression and relapse offer personalized treatment options for preventing recurrence. By inhibiting key molecules involved in critical signaling pathways, such as the HER2 receptor in breast cancer or the BRAF protein in melanoma, targeted therapies can effectively suppress tumor growth and reduce the risk of relapse [383, 384]. Immunotherapy has emerged as a promising approach for preventing relapse by harnessing the patient’s immune system to recognize and eliminate residual cancer cells [385, 386]. Checkpoint inhibitors, adoptive cell therapies, and cancer vaccines aim to boost immune responses against tumor cells, preventing their survival and proliferation. Furthermore, lifestyle modifications and supportive care interventions play a critical part in preventing cancer relapse. These may encompass dietary changes, exercise programs, stress management techniques, and psychosocial support to promote overall health and well-being, reduce the risk of recurrence, and improve treatment outcomes [387, 388].

Combination therapies represent a promising approach to address tumor dormancy and relapse by targeting multiple aspects of the disease simultaneously. One strategy involves combining traditional treatments, such as chemotherapy or radiation therapy, with targeted agents that specifically inhibit molecular pathways implicated in tumor progression and relapse [389]. For example, combining chemotherapy with targeted therapies that block survival pathways, such as PI3K/Akt or MAPK/ERK, can enhance treatment efficacy by preventing the survival and reactivation of dormant cancer cells [231, 390]. Immunotherapy can also be integrated into combination treatment strategies to leverage the patient’s immune system against cancer cells [391, 392]. Combining checkpoint inhibitors with targeted therapies or traditional treatments may improve immune responses and enhance tumor control, reducing the risk of relapse. Furthermore, integrating multiple targeted agents with complementary mechanisms of action can overcome treatment resistance and heighten therapeutic efficacy. By targeting different molecular pathways simultaneously, combination therapies can disrupt the complex network of signaling pathways driving tumor growth and relapse. Additionally, combining therapeutic approaches targeting cancer cells and the TME can improve treatment outcomes [393]. Moreover, incorporating precision medicine approaches, such as genetic profiling and liquid biopsies, into combination treatment strategies facilitate personalized treatment selection and monitoring of treatment response. By tailoring therapies to the tumor’s specific molecular characteristics and adjusting treatment regimens based on real-time biomarker data, clinicians can optimize treatment outcomes and minimize the risk of relapse.

## Challenges and future directions

This review tackles challenges and future directions in understanding and managing tumor dormancy and relapse. One challenge is the heterogeneity of dormant cancer cells, which may exhibit diverse molecular profiles and responses to therapy, making it difficult to develop targeted treatments [394, 395]. Another challenge lies in the complexity of the TME, which plays a crucial role in regulating dormancy and relapse [58, 396]. Comprehending the dynamic interactions between cancer cells and the microenvironment is essential for developing effective therapeutic strategies. Moreover, the lack of reliable biomarkers for detecting dormant cancer cells and predicting relapse poses a significant challenge. Identifying molecular markers and imaging techniques capable of accurately detecting minimal residual disease and predicting recurrence risk is critical for improving patient outcomes. Furthermore, therapeutic resistance remains a major obstacle in preventing tumor relapse. Cancer cells can develop resistance to therapy through various mechanisms, including genetic mutations, epigenetic alterations, and changes in the TME. Overcoming resistance requires innovative treatment approaches and combination therapies targeting multiple pathways. Additionally, the role of the immune system in regulating tumor dormancy and relapse presents challenges and opportunities. While the immune system can suppress dormant cancer cells and prevent relapse, it can also promote tumor growth and metastasis under certain conditions. Understanding the complex interplay between cancer cells and the immune system is essential for developing immunotherapeutic strategies to prevent relapse.

Future directions for research in tumor dormancy and relapse include advancing our comprehension of the molecular mechanisms underlying dormancy, identifying novel therapeutic targets, and developing innovative treatment strategies. Integrating multidisciplinary approaches, such as genomics, immunology, and systems biology, will be pivotal for unraveling the intricacies of tumor dormancy and relapse. Furthermore, capitalizing on emerging technologies, such as single-cell analysis, liquid biopsies, and advanced imaging techniques, holds promise for improving early detection, monitoring treatment response, and predicting recurrence risk.

## Conclusions

In conclusion, this review comprehensively examines the complex processes underlying tumor dormancy and relapse. By exploring various hallmarks such as cellular dormancy, angiogenic dormancy, and immunologic dormancy, as well as the role of TME, a refined understanding of tumor recurrence emerges. Despite considerable progress in detecting minimal residual disease and predicting relapse risk, challenges remain, including the heterogeneity of dormant cancer cells and the deficiency of reliable biomarkers. Nevertheless, therapeutic strategies targeting dormant cells and preventing relapse hold potential, with combination therapies and immunotherapy presenting new avenues for intervention. Future directions in research emphasize the necessity for interdisciplinary collaboration, innovative methodologies, and continuous translational efforts to fully unravel the complexities of tumor recurrence. By addressing these challenges and embracing future directions, we can strive towards more effective strategies for preventing relapse and improving the outcomes of patients with cancer worldwide.

## Data Availability

Not applicable.

## Abbreviations

APC: Adenomatous polyposis coli
BRAF: B-Raf proto-oncogene, serine/threonine kinase
2-HG: 2-Hydroxyglutarate
CAR T: Chimeric antigen receptor T
CAFs: Cancer-associated fibroblasts
cfDNA: Cell-free DNA
CSCs: Cancer stem cells
CT: Computed tomography
CTCs: Circulating tumor cells
CTLs: Cytotoxic T lymphocytes
CTLA-4: Cytotoxic T-lymphocyte-associated protein 4
ctDNA: Circulating tumor DNA
DCE-MRI: Dynamic contrast-enhanced MRI
DTCs: Disseminated tumor cells
DWI: Diffusion-weighted imaging
ECM: Extracellular matrix
EMT: Epithelial-to-mesenchymal transition
EVs: Extracellular vesicles
FAK: Focal adhesion kinase
FGF: Fibroblast growth factor
G-CSF: Granulocyte colony-stimulating factor
HDACs: Histone deacetylation
HER2: Human epidermal growth factor receptor 2
HIFs: Hypoxia-inducible factors
IFN-γ: Interferon-γ
IL: Interleukin
KRAS: Kirsten rat sarcoma virus
lncRNAs: Long non-coding RNAs
MDSCs: Myeloid-derived suppressor cells
MEK: Mitogen-activated protein kinase kinase
miRNAs: MicroRNAs
mTORC2: MTOR complex 2
HNSCC: Head and neck squamous cell carcinoma
MRI: Magnetic resonance imaging
MSCs: Mesenchymal stem cells
NETs: Neutrophil extracellular traps
NGS: Next-generation sequencing
NK: Natural killer
PD-L1: Programmed death-ligand 1
PET: Positron emission tomography
PI3K: Phosphoinositide 3-kinase
PIP2: Phosphatidylinositol 4,5-bisphosphate
PIP3: Phosphatidylinositol 3,4,5-trisphosphate
PTEN: Phosphatase and tensin homolog
RTKs: Receptor tyrosine kinases
SCID: Severe combined immunodeficient
TCF/LEF: T cell factor/lymphoid enhancer-binding factor
TGF-β: Transforming growth factor-β
TME: Tumor microenvironment
Tregs: Regulatory T cells
TSP-1: Thrombospondin-1
VEGF: Vascular endothelial growth factor
